# Interfacial Synergy in a Band‐Aligned Low‐Dimensional Heterojunction Toward Broadband Photodetection

**DOI:** 10.1002/advs.202520919

**Published:** 2026-02-26

**Authors:** Yuanfeng Wen, Kening Xiao, Yao Yang, Mengjie Jiang, Libo Zhang, Shicong Hou, Hang Ma, Yunduo Zhang, Wenqi Mo, Yiran Tan, Bolang Peng, Jinqiu Huang, Xueyuan Wei, Chunming Liu, Bangjian Zhao, Jiale He, Qing Li, Songyuan Ding, Yi Zhou, Guanhai Li, Xiaoshuang Chen

**Affiliations:** ^1^ College of Physics and Optoelectronic Engineering Hangzhou Institute for Advanced Study University of Chinese Academy of Sciences Hangzhou P. R. China; ^2^ College of Physics and Optoelectronic Engineering Shenzhen University Shenzhen P. R. China; ^3^ Shanghai Key Lab of Modern Optical System University of Shanghai for Science and Technology Shanghai P. R. China; ^4^ Shanghai Youhe Information Technology Co., Ltd. Shanghai China; ^5^ State Key Laboratory of Infrared Physics Shanghai Institute of Technical Physics Chinese Academy of Sciences Shanghai P. R. China; ^6^ Suzhou Laboratory Suzhou Jiangsu P. R. China

**Keywords:** broadband photodetector, heterojunction, infrared and terahertz, logic gates

## Abstract

Monolithic integration of sensing, imaging, and communication from visible to terahertz bands on a single chip remains challenging as efficient detection across such disparate energies demands stringent, often incompatible material properties. However, conventional semiconductors suffer from intrinsic bandgap limitations or require cryogenic cooling, while existing low‐dimensional heterojunctions often struggle to synergize multiband response kinetics and suppress interfacial carrier recombination. Here, we demonstrate a band‐aligned low‐dimensional heterojunction based on Ta_2_NiSe_5_ and Sb_2_Te_3_ that leverages interfacial synergy to unify complementary photoresponse pathways, thereby enabling ultrabroadband and low‐noise photodetection spanning across visible to terahertz regimes. The device delivers stable responsivities of up to 0.19 A·W^−1^ in the visible–near‐infrared regime, while antenna‐enhanced coupling extends detection into the terahertz range with microsecond transients, 10 kHz bandwidth, and a minimum noise‐equivalent power of 26 pW·Hz^−1/2^. A built‐in potential of 115 meV, originating from the Type‐II band alignment, plays a dual role by ensuring  highly efficient carrier separation in the short‐wavelength band and  driving a strong photothermoelectric effect in the terahertz regime. The synergistic interfacial response allows further implementation of reconfigurable logic operations and dual‐channel ASCII data transmission using visible and terahertz signals as independent carriers, highlighting its potential for anti‐jamming communication and multifunctional photonic systems.

## Graphical abstract

Monolithically integrating sensing and communication, a Ta_2_NiSe_5_/Sb_2_Te_3_ heterojunction achieves visible‐to‐near‐infrared photovoltaics with high‐speed terahertz photo‐thermoelectric detection at room temperature. By engineering a Type‐II van der Waals heterojunction, we overcome the intrinsic spectral limitations and disparate physical requirements of conventional broadband photodetectors. This synergistic platform further enables reconfigurable logic and dual‐channel ASCII transmission, paving the way for anti‐jamming communication and multifunctional photonic systems.

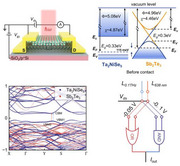

## 1 Introduction

The ability to seamlessly perceive and process optical information from the visible (VIS) to the terahertz (THz) spectrum on a single, compact platform is a defining objective for next‐generation technologies, poised to revolutionize fields from multi‐spectral imaging and non‐destructive sensing to high‐bandwidth secure communications [1, 2, 3, 4]. As these fields advance, there is a growing demand for photodetectors capable of operating across a broad spectrum—from VIS to THz frequencies—with high sensitivity, fast response, low noise, and excellent signal‐to‐noise ratio. Such performance is essential to support real‐time, high‐fidelity signal acquisition and processing in next‐generation integrated photonic platforms [5].

Photodetectors can be categorized into photon‐type and thermal‐type detectors based on the disparate light‐induced physical processes involved. Photon‐type detectors transform photons into electrical signals directly, demonstrating attributes such as high‐speed operation, excellent sensitivity, and precise spatial resolution. For example, InGaAs can also achieve a specific detectivity of 10^12^ Jones and a responsivity of 0.8 A·W^−1^ in the 1.5 µm communication band, which is an order of magnitude higher than the old Ge detector [6]. At 77 K, the InSb detector has a *D* ^*^ of 2.2 × 10^11^ Jones and a NEP of ∼5 × 10^−12^ W·Hz^−1/2^ [7]. At 77 K operating temperature, the HgCdTe detector has a responsivity of ∼2 A·W^−1^, a specific detectivity *D* ^*^ of ∼2 × 10^11^ Jones, a NEP of ∼1 × 10^−11^ W·Hz^−1/2^, and a response time of <1 µs in the 5 µm band [8]. Nevertheless, the photon‐type photodetectors' photocurrent response is limited by the band gap of the material and requires a complex generation process and low temperature cooling of liquid nitrogen, resulting in limited application. Thermal‐type photodetectors generate electrical signals by absorbing the change of electron or lattice temperature caused by the thermalization of carriers after photon radiation. The thermal detector can realize mid‐wave infrared and even long‐wave infrared detection at room temperature due to its wavelength‐insensitive characteristics and uncooled working mode. For example, the VO_x_ detector can achieve 8–14 µm broadband detection at room temperature, NEP as low as 1 × 10^10^ W·Hz^−1/2^, response time ∼10 ms [9]. ZnTe photodetector can achieve broadband response from visible to near infrared (VIS–NIR) region, the responsivity is 18.3 A·W^−1^ and *D* ^*^ is 2.89 × 10^9^ Jones at 405 nm [10]. Nevertheless, the thermal‐type photodetectors are limited by the response mechanism in sensitivity, response time, and NEP, so there is a limitation in fast response and real‐time imaging.

Therefore, a paradigm shift is urgently needed toward novel material platforms and device architectures that can circumvent these intrinsic limitations, enabling room‐temperature, high‐performance photodetection across the entire VIS‐to‐THz spectrum within a single, scalable device. Compared to traditional bulk semiconductors, low‐dimensional materials offer unique advantages for broadband optoelectronics, including wide spectral absorption, mechanically flexible integrability, layer‐tunable band gaps, strong quantum confinement, and facile integration into chip‐scale architectures [11, 12, 13, 14]. These attributes have spurred intensive exploration of 2D materials for photodetection, where rational material design and device engineering have been widely employed to extend spectral coverage and enhance responsivity. These include device‐level innovations such as electrostatic gating, integration of plasmonic nanostructures, and the construction of van der Waals heterostructures, as well as material‐level band engineering through defect introduction, atomic doping, vacancy formation, and grain boundary control [15, 16, 17, 18, 19, 20]. Such approaches effectively modulate electronic and optical characteristics, enabling photodetection across ultraviolet to terahertz frequencies. While diverse 2D materials have been explored for broadband optoelectronics—including transition metal dichalcogenides (MoS_2_ [21], WS_2_ [22], WSe_2_ [23] and MoTe_2_ [24] with layer‐tunable bandgaps), black phosphorus (BP with high mobility and anisotropic absorption) [25], graphene (gapless and ultrafast but with weak intrinsic responsivity) [26], topological insulators (Bi_2_Se_3_ and Sb_2_Te_3_ with spin–momentum–locked surface states) [27, 28], and emerging MXenes or perovskite‐derived sheets [29] and 2D semimetals (NiTe_2_, MoTe_2_, PdTe_2_, PtTe_2_) with gapless band structures, high carrier mobility, and topological surface states—recent breakthroughs have highlighted 2D semimetals as game‐changers for broadband detection [30]. Via low‐temperature (300–400°C) wafer‐scale synthesis compatible with CMOS back‐end‐of‐line processes, these materials enable the construction of mixed‐dimensional vdW heterostructures (NiTe_2_/Si [31], 1T′‐MoTe_2_/Si [32], PdTe_2_/Si [33]) that achieve ultrabroadband response spanning UV (265 nm) to long‐wave infrared (10.6 µm) at room temperature. These devices deliver exceptional performance metrics, including high responsivity (up to 682.7 mA·W^−^^1^ at 980 nm), ultralow dark current (3.4 × 10^−^^14^ A), specific detectivity exceeding 10^10^ Jones in mid‐infrared regions, and fast response speeds (1.5–41.5 µs), while supporting self‐powered operation through photovoltaic effects [31, 32, 33]. Their working mechanisms integrate multiple photoresponse pathways—photovoltaic effect for short wavelengths, internal photoemission and photo‐thermionic emission for infrared—enabled by Type‐II band alignment and strong built‐in electric fields at heterojunctions, overcoming the single‐mechanism limitation of conventional 2D semiconductors. These intrinsic bottlenecks—including the bandgap rigidity and weak optical coefficients of TMDs, the air‐sensitivity of BP and encapsulation‐induced attenuation of THz response, the atomically thin absorption and gain dependence of graphene despite ultrafast transport, the dominance of bulk carriers and Fermi‐level pinning in topological insulators that obscure surface‐state transport and raise dark current, the metallic conductivity and limited built‐in fields of MXenes that lead to high Johnson noise, and the instability and out‐of‐plane transport bottlenecks of perovskite‐derived sheets—prevent any single 2D semiconductor from unifying photoconductive (VIS–NIR) and photothermoelectric (THz) mechanisms. Unlike traditional epitaxial heterojunctions constrained by lattice matching requirements (which limit material combinations), van der Waals (vdW) heterostructures rely on weak interlayer van der Waals forces for assembly, enabling seamless integration of dissimilar low‐dimensional materials (2D, quasi‐1D) with complementary optical/electronic properties—this interface‐free lattice mismatch characteristic is foundational to their ability to tailor multiband photoresponse mechanisms.

In this work, we report the design and realization of a van der Waals heterojunction that integrates a band‐aligned layered semiconductor with an air‐stable topological material, thereby enabling room‐temperature broadband photodetection spanning the VIS–NIR to THz regimes. The study designs a quasi‐1D/2D van der Waals (vdW) heterojunction by integrating Ta_2_NiSe_5_ (quasi‐1D, narrow bulk bandgap of 0.33 eV and high carrier mobility > 556.5 cm^2^·V^−1^·s^−1^) [34] and Sb_2_Te_3_ (2D, strong Seebeck coefficient ≈ 164 µV·K^−1^ and intrinsic THz absorption) [35]. The heterostructure leverages a Type‐II band alignment that creates a strong built‐in electric field, enabling efficient photocarrier separation and dual‐mechanism responsivity, in which photoconduction effect dominates from VIS–NIR, yielding a high responsivity of 0.11 A·W^−1^ at 638 nm and fast response down to 5.9 ms, while the photothermoelectric effect drives zero‐bias THz detection at 0.1 THz, facilitated by an integrated bow‐tie antenna, with ultrafast response up to 4.38 µs. By synergistically combining spectrally complementary mechanisms in a stable, miniaturizable architecture, this work establishes a new platform for high‐sensitivity, wide‐band photodetection and provides a generalizable strategy for multi‐spectral optoelectronic systems.

## 2 Results and Discussion

**FIGURE 1:**
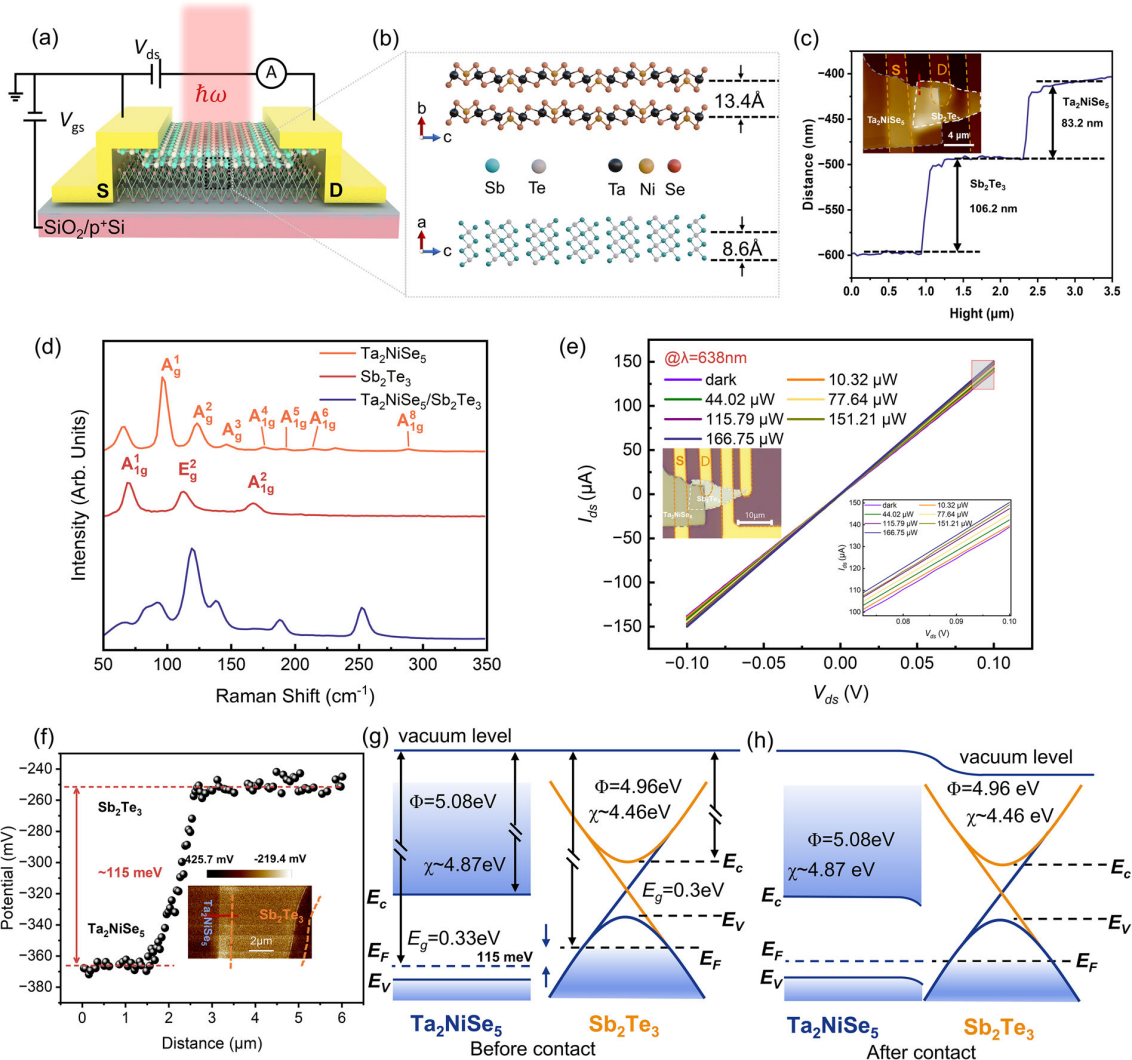
Characterization of Ta_2_NiSe_5_/Sb_2_Te_3_ heterostructure on the SiO_2_/p+Si substrate. (a) A structural diagram of the Ta_2_NiSe_5_/Sb_2_Te_3_ van der Waals heterojunction photodetector. (b) Ta_2_NiSe_5_ and Sb_2_Te_3_ atomic structure amplification diagram. (c) Morphology of the heterojunction region and the corresponding thickness measured along the white dashed line. (d) Raman spectra corresponding to Ta_2_NiSe_5_, Sb_2_Te_3_ flakes, and the heterojunction region. The spectra are labeled according to their Raman peaks. (e) *I*_ds_‐*V*_ds_ curves at different powers under 638 nm laser irradiation, the inset shows the optical image of the Ta_2_NiSe_5_/Sb_2_Te_3_ heterojunction. (f) The contact potential difference curve between Ta_2_NiSe_5_ and Sb_2_Te_3_ extracted along the red line. The Inset image is the KPFM image of the Ta_2_NiSe_5_/Sb_2_Te_3_ heterojunction photodetector. (g, h) The band alignment between Sb_2_Te_3_ and Ta_2_NiSe_5_ before and after contact. *E*_c_ (Sb_2_Te_3_) = −4.467 eV, *E*_v_ (Sb_2_Te_3_) = −4.767 eV, *E*_c_ (Ta_2_NiSe_5_) = −4.842 eV, *E*_v_ (Ta_2_NiSe_5_) = −5.202 eV.

To explore the broadband photoresponse, a vertically stacked 2D Ta_2_NiSe_5_/Sb_2_Te_3_ heterostructure was assembled onto the SiO_2_/p^+^Si substrate using mechanical exfoliation together with a dry transfer method, as depicted in Figure 1a. The detailed atomic structures of the Ta_2_NiSe_5_ and Sb_2_Te_3_ flakes are shown in Figure 1b, where the distance between the two atomic layers of Ta_2_NiSe_5_ is 13.4 Å, and the distance between the same atoms in the Sb_2_Te_3_ lattice is 8.6 Å, with an 8.6 µm wide overlap region. In this experiment, the overlap region of Ta_2_NiSe_5_/Sb_2_Te_3_ heterojunction is located at the center of two metal electrodes by dry transfer technology, which effectively reduces the lateral transmission distance of photogenerated carriers and alleviates the influence of series resistance. Atomic force microscopy (AFM) characterization in Figure 1c reveals that the Ta_2_NiSe_5_ and Sb_2_Te_3_ sheets exhibit uniform surface terraces with average thicknesses of ≈ 83.2 nm and ≈ 106.2 nm, respectively, thereby confirming the successful deposition of continuous layers suitable for constructing high‐quality heterojunction devices. The assembly of the Ta_2_NiSe_5_ and Sb_2_Te_3_ heterostructure is confirmed through Raman spectroscopy, as shown in Figure 1d. The Raman spectrum exhibits characteristic peaks of both Ta_2_NiSe_5_ and Sb_2_Te_3_, with a notable weakening of these peaks in the overlap region (indicated by the blue solid line), which indicates that the heterojunction has a high interfacial quality with strong interlayer coupling effects [36]. The Raman modes for Ta_2_NiSe_5_ were assigned, and seven Raman peaks were identified. The Raman active modes of 96.1 cm^−1^, 123.4 cm^−1^, 146.4 cm^−1^, 175.5 cm^−1^, 192.2 cm^−1^, and 212.9 cm^−1^ are assigned to A_g_^1^ to A_g_^6^, respectively. The mode of 289 cm^−1^ is assigned to A_g_^8^, which is consistent with a previous report [37]. Additionally, the peaks at 68.7 cm^−1^ (out‐of‐plane vibration mode A_1g_^1^), 112.9 cm^−1^ (in‐plane vibration mode E_g_^2^), and 167.2 cm^−1^ (out‐of‐plane vibration mode A_1g_^2^) are attributed to Sb_2_Te_3_, in agreement with the previous reports [28]. Under dark conditions, the current–voltage (*I*–*V*) characteristics of the heterostructure (Figure 1e) display a highly linear dependence of current on drain–source bias (*V*_ds_), indicative of pronounced ohmic behavior that thereby excluding significant contact resistance at the metal–semiconductor interfaces and thereby ensures that subsequent photocurrent responses can be ascribed to intrinsic heterojunction properties rather than extrinsic electrode effects, while under laser illumination the extracted photocurrent (*I*_ph_ = *I*_light_ – *I*_dark_) increases linearly with incident optical power, further corroborating the stability and reliability of charge transport across the junction. To further elucidate the interfacial energetics that govern the photoresponse of the Ta_2_NiSe_5_/Sb_2_Te_3_ heterojunction, Kelvin probe force microscopy (KPFM) was employed to directly probe the local surface potential distribution. As displayed in Figure 1f, 2D KPFM mapping clearly resolves a step‐like contrast at the interface, corresponding to a contact potential difference of 115 meV. This potential offset evidences the presence of a built‐in barrier, which originates from the slight difference in work functions between Ta_2_NiSe_5_ [38] (4.967 eV) and Sb_2_Te_3_ (5.082 eV) [39]. Guided by these measurements and calculations and consistent with previous photoemission reports [40, 41, 42], the corresponding band alignment was constructed before and after junction formation (Figure 1g,h), revealing a staggered Type‐II configuration. Upon contact and reaching thermal equilibrium, the Fermi levels align, inducing downward band bending in Ta_2_NiSe_5_ and upward bending in Sb_2_Te_3_. This band structure is fundamentally advantageous for photodetection, as it creates an energetically favorable cascade for the spatial separation of photogenerated carriers—confining electrons to the Ta_2_NiSe_5_ side and holes to the Sb_2_Te_3_ side. The interfacial field not only promotes spatial charge separation but also suppresses carrier recombination, thereby providing the physical basis for the broadband photodetection capability observed in the device. With the interfacial electronic structure established, we employed scanning photocurrent microscopy (SPCM) to elucidate the bias‐dependent carrier transport mechanisms.

**FIGURE 2:**
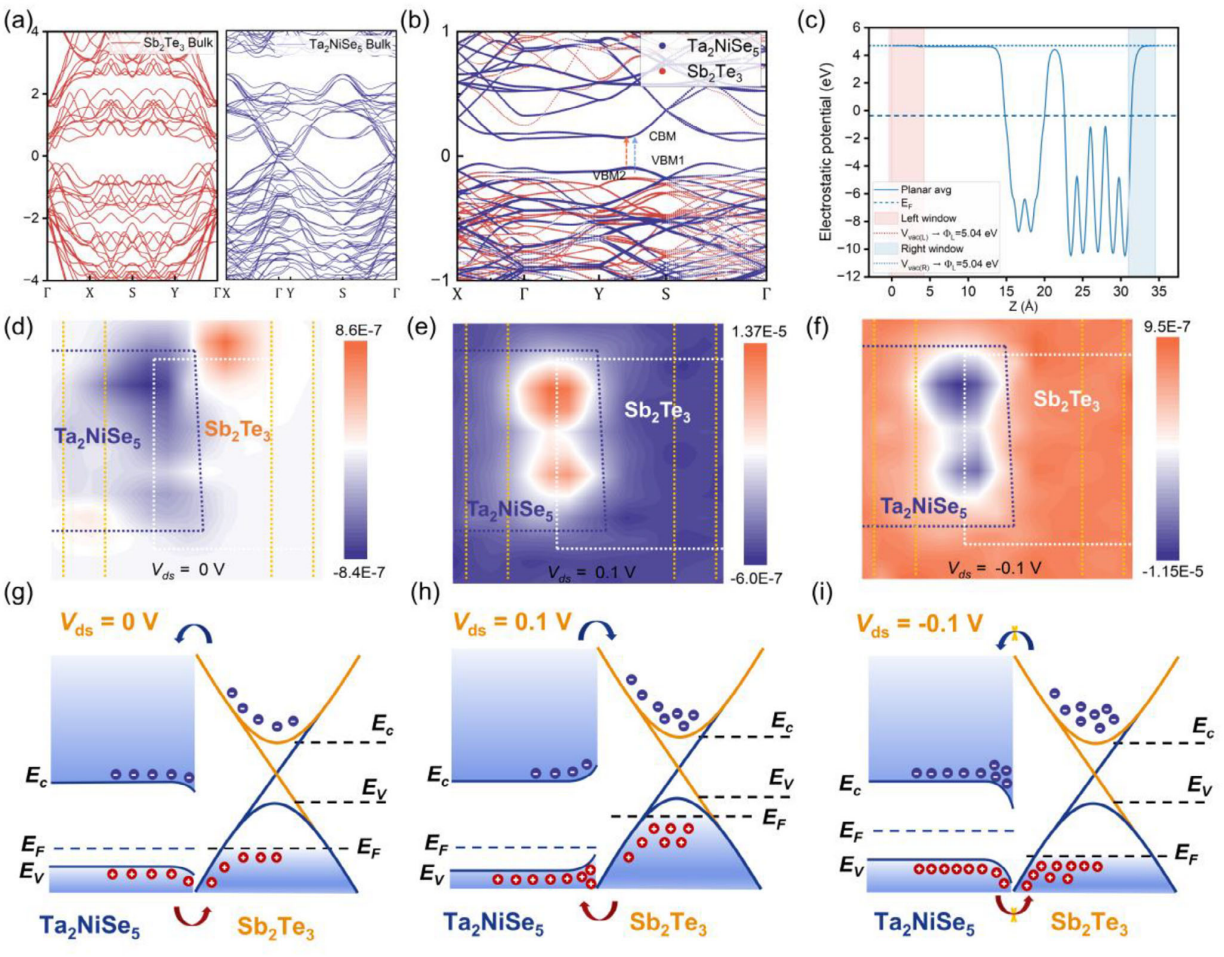
Electronic band analysis of Ta_2_NiSe_5_/Sb_2_Te_3_ van der Waals heterojunction. (a) Band structures of Ta_2_NiSe_5_/Sb_2_Te_3_ heterojunction calculated using DFT. (b) Partial charge density of Ta_2_NiSe_5_/Sb_2_Te_3_ heterojunction associated with the VBM and CBM states. (c) The planar averaged electrostatic potential of Ta_2_NiSe_5_/Sb_2_Te_3_ heterojunction plane calculated using DFT. (d–f) Photocurrent mapping images measured at 0 V, 0.1 V, and −0.1 V bias under 638 nm laser illumination. (g–i) Band alignment of Ta_2_NiSe_5_/Sb_2_Te_3_ heterojunction under zero, forward, and reverse bias under laser illumination.

To better understand the electronic characteristics of the Ta_2_NiSe_5_/Sb_2_Te_3_ heterostructure, we first calculated its band structure, as shown in Figure 2a,b. The projected band dispersions reveal that the states near the Fermi level are mainly contributed by Ta_2_NiSe_5_ (blue) with substantial hybridization from Sb_2_Te_3_ (red), indicating strong interfacial coupling. Notably, the conduction band minimum (CBM) of Ta_2_NiSe_5_ is aligned close to the valence band maximum (VBM) of Sb_2_Te_3_, forming a staggered Type‐II band alignment. Such band alignment facilitates spatial separation of photogenerated carriers, where electrons are preferentially localized in Ta_2_NiSe_5_ while holes are confined in Sb_2_Te_3_. This band configuration is particularly advantageous for infrared photodetection, as the reduced recombination probability significantly extends carrier lifetime and improves photoresponse efficiency. Moreover, the relatively narrow effective bandgap in the vicinity of the Γ point suggests potential absorption in the near‐infrared regime, which can be further tuned by external bias or electrostatic gating. The electrostatic potential profile across the heterostructure (Figure 2c) provides additional evidence of interfacial charge redistribution. The planar‐averaged potential shows a well‐defined vacuum level on both sides, yielding a consistent work function of 5.04 eV. Within the heterostructure region (15–25 Å), pronounced oscillations are observed, reflecting interlayer interactions and charge transfer.

The built‐in electric field generated at the interface is expected to facilitate efficient separation and transport of photogenerated carriers, thereby enabling photovoltaic‐type operation without external bias. This self‐driven mechanism not only enhances carrier collection but also suppresses dark current, thereby improving the detectivity of the device. Without any applied bias, the device response is governed predominantly by its intrinsic built‐in fields; the photoresponse manifests as a photovoltaic effect spatially confined to the interfacial region (Figure 2d), a direct consequence of the built‐in electrostatic potential gradient efficiently dissociating photogenerated excitons and sweeping the resultant electrons and holes into the Ta_2_NiSe_5_ and Sb_2_Te_3_ channels, respectively. The application of forward bias induces a complete polarity inversion of the photocurrent (Figure 2e), serving as unambiguous evidence of a fundamental shift from an internal‐field‐driven photovoltaic mechanism to an externally modulated transport regime. In this case, the forward bias progressively cancels the built‐in potential (Figure 2h), such that the net photoresponse becomes dominated by the photomodulation of the junction's forward *I–V* characteristics, with both the direction and amplitude of the photocurrent dictated by the externally applied bias. Under an applied reverse bias (*V*_ds_ = −0.1 V), the device exhibits a significantly enhanced photoresponse (Figure 2f). The external potential superimposes constructively with the built‐in field (Figure 2i), leading to two synergistic effects: (i) the depletion region widens, expanding the active volume for photocarrier generation and collection; and (ii) the magnitude of the junction electric field increases, which accelerates carrier drift velocity. The resulting reduction in carrier transit time across the junction drastically mitigates recombination losses, leading to near‐unity carrier collection efficiency and a substantially amplified photocurrent.

**FIGURE 3:**
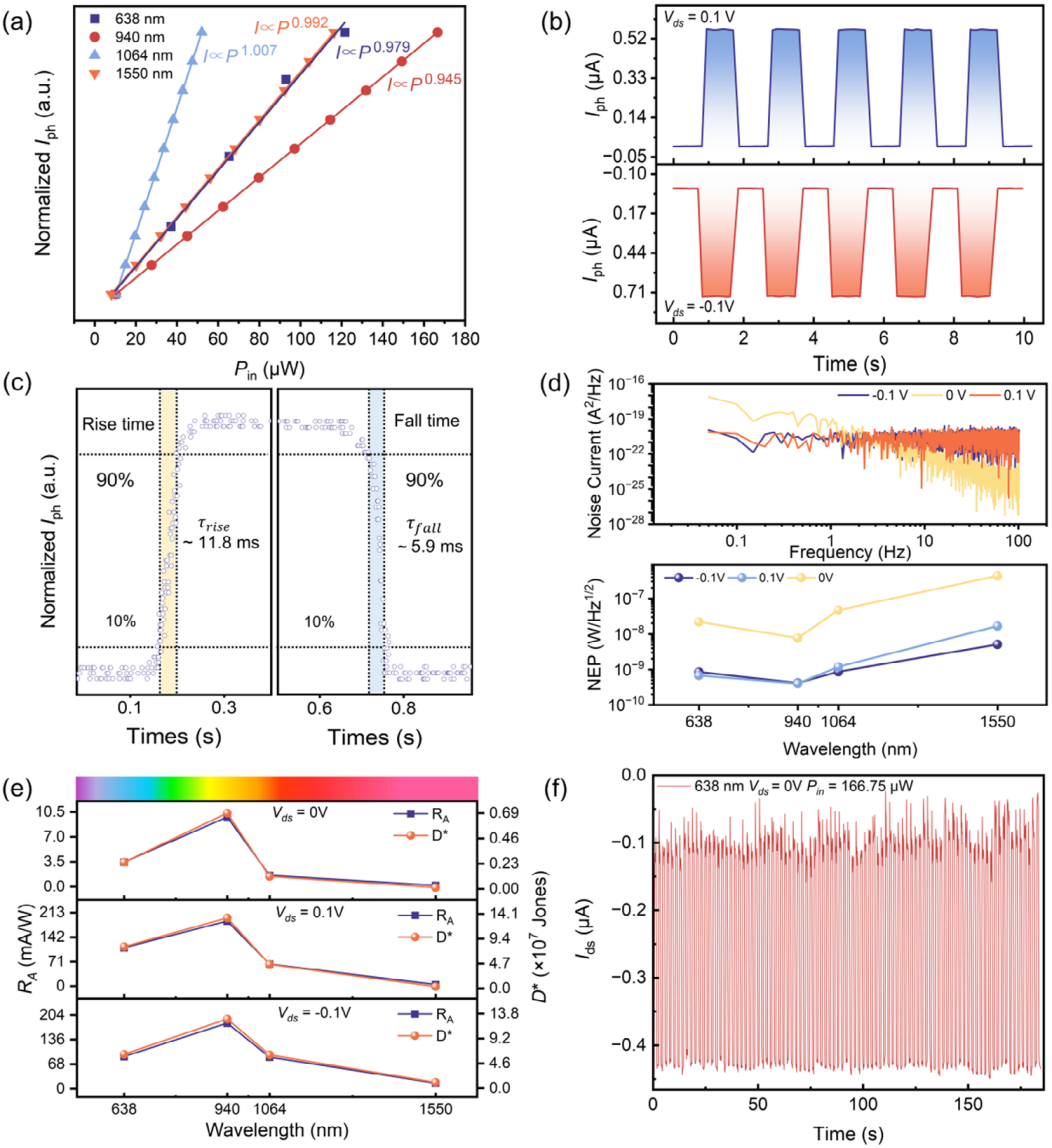
The VIS–NIR light response characteristics of Ta_2_NiSe_5_/Sb_2_Te_3_ van der Waals heterojunction. (a) The fitting photocurrent versus laser incident power of the device under different‐wavelength light illumination at 0.1 V bias by the power law. (b) The optical switching characteristics were measured under 1550 nm laser illumination with an incident power of 116 µW under different bias voltages. (c) Ta_2_NiSe_5_/Sb_2_Te_3_ photodetector, the rising and falling edge of the single‐cycle normalized response curve at 638 nm. (d) The photodetector current noise power spectrum at *V*_ds_ = −0.1 V, 0 V, 0.1 V, and the NEP at different wavelengths. (e) *R*_A_ and *D* ^*^ under different wavelengths of 638–1550 nm at 0 V, 0.1 V, −0.1 V bias, and 10 µW fixed incident power. The single photon energies at 638 nm, 940 nm, 1064 nm, and 1550 nm are 1.94 eV, 1.32 eV, 1.17 eV, and 0.80 eV, respectively. (f) Long‐time photoresponse reproducibility of Ta_2_NiSe_5_/Sb_2_Te_3_ photodetector at fixed incident power.

The broadband photodetection characteristics of the Ta_2_NiSe_5_/Sb_2_Te_3_ heterojunction were systematically evaluated under VIS–NIR illumination. The response time, external quantum efficiency (EQE), noise equivalent power (NEP), responsivity (*R*_A_), and specific detectivity (*D* ^*^) are the core indicators used to evaluate the performance of photodetectors [43]. The dependence of the photocurrent (*I*_ph_) on incident optical power (*P*) is a critical parameter that defines the detector's linear dynamic range (LDR). A linear laser‐power‐dependent photocurrent behavior (*I*_ph_ ∼ A*P* ^α^) is observed in Figure 3a, where the values of α are 0.979, 0.945, 1.007, and 0.992, respectively, which can be attributed to the complex interaction of electron‐hole pair generation, recombination, and trapping dynamics in the heterojunction [44, 45]. The linear dependence confirms that the photocurrent is directly proportional to the absorbed photon flux, demonstrating the device's suitability for quantitative photodetection across variable illumination conditions.

Figure 3b presents the photocurrent switching response under 1550 nm excitation, a telecommunication‐relevant wavelength, where the device exhibits highly repeatable ON/OFF transients with minimal baseline drift. The robustness of the signal at this wavelength underscores the efficient carrier separation sustained by the interfacial band alignment, even at low photon energies. The response time of photodetectors is investigated by collecting a cycle of ON/OFF states under a modulated optical signal with an oscilloscope.

The rise time and fall time are usually defined as the time required for the photocurrent to increase from 10% to 90% and to decrease from 90% to 10% of the maximum photocurrent, respectively. Figure 3c shows the rising and falling edges of the time‐resolved light response extracted from a single period, indicating that the rising time is ∼11.8 ms and the falling time is ∼5.9 ms. It should be noted that these response times are primarily limited by the experimental low‐modulation‐frequency laser sources and bandwidth constraints of the measurement electronics, rather than the intrinsic carrier dynamics of the heterojunction. As discussed in the mechanism section, the high carrier mobility of Ta_2_NiSe_5_ and the Type‐II band alignment‐induced built‐in electric field are expected to support much faster intrinsic carrier separation and transport. Figure 3d (top panel) presents the noise power spectrum across frequencies (0.1–100 Hz) at three bias conditions (*V*_ds_ = −0.1 V, 0 V, 0.1 V). In the high‐frequency regime (>1000 Hz), white noise (dominated by Johnson and shot noise) is prominent, while 1/*f* noise contributes at low frequencies (<1000 Hz). The NEP under different bias voltages and spectral bands is shown in Figure 3d. The NEP at 0.1 V bias from 638 nm to 1550 nm shows a slight fluctuation from 0.67 nW·Hz^−1/2^ to 16.5 nW·Hz^−1/2^. The NEP under 0.1 V bias is lower than that under 0 V bias, indicating that the device works under bias and exhibits superior performance . As shown in Figure 3e, the wavelength‐dependent responsivity and specific detectivity at different bias voltages are calculated to evaluate the photoresponse performance of the photodetector in the VIS and NIR regions. At a bias voltage of 0.1 V, the responsivities at wavelengths of 638 nm, 940 nm, 1064 nm, and 1550 nm are 0.11 A·W^−1^, 0.19 A·W^−1^, 0.064 A·W^−1^, and 0.0045 A·W^−1^, respectively.

The heterojunction photodetector achieves a photosensitive peak at 940 nm, yielding a photoresponsivity of 0.19 A·W^−1^, and a specific detectivity of 1.33 × 10^8^ Jones. The dynamic responsivity varying with the incident light power is shown in Figure S7. From the figure, the responsivity curve is mainly linear, and the fluctuation is small, which proves the excellent linear dynamic stability of the photodetector. Figure S8 shows the *D* ^*^ and EQE of the photodetector at different wavelengths at *V*_ds_ = 0.1 V with the change of incident light power. The *D* ^*^ and EQE at 638 nm and 940 nm decrease with the increase of light intensity; this decrease at high light intensity is ascribed to enhanced carrier recombination by defects [24]. Figure 3f shows the long‐term optical response repeatability test under periodic optical modulation at fixed incident power. The device exhibits highly stable and reproducible ON/OFF switching over a continuous 180 s operation window, with negligible baseline drift or signal degradation. The persistence of this stable photoresponse underscores the robustness of the Ta_2_NiSe_5_/Sb_2_Te_3_ heterojunction against photoinduced fatigue and thermal perturbations, thereby validating its operational reliability for sustained broadband detection from the VIS–NIR regime.

**FIGURE 4:**
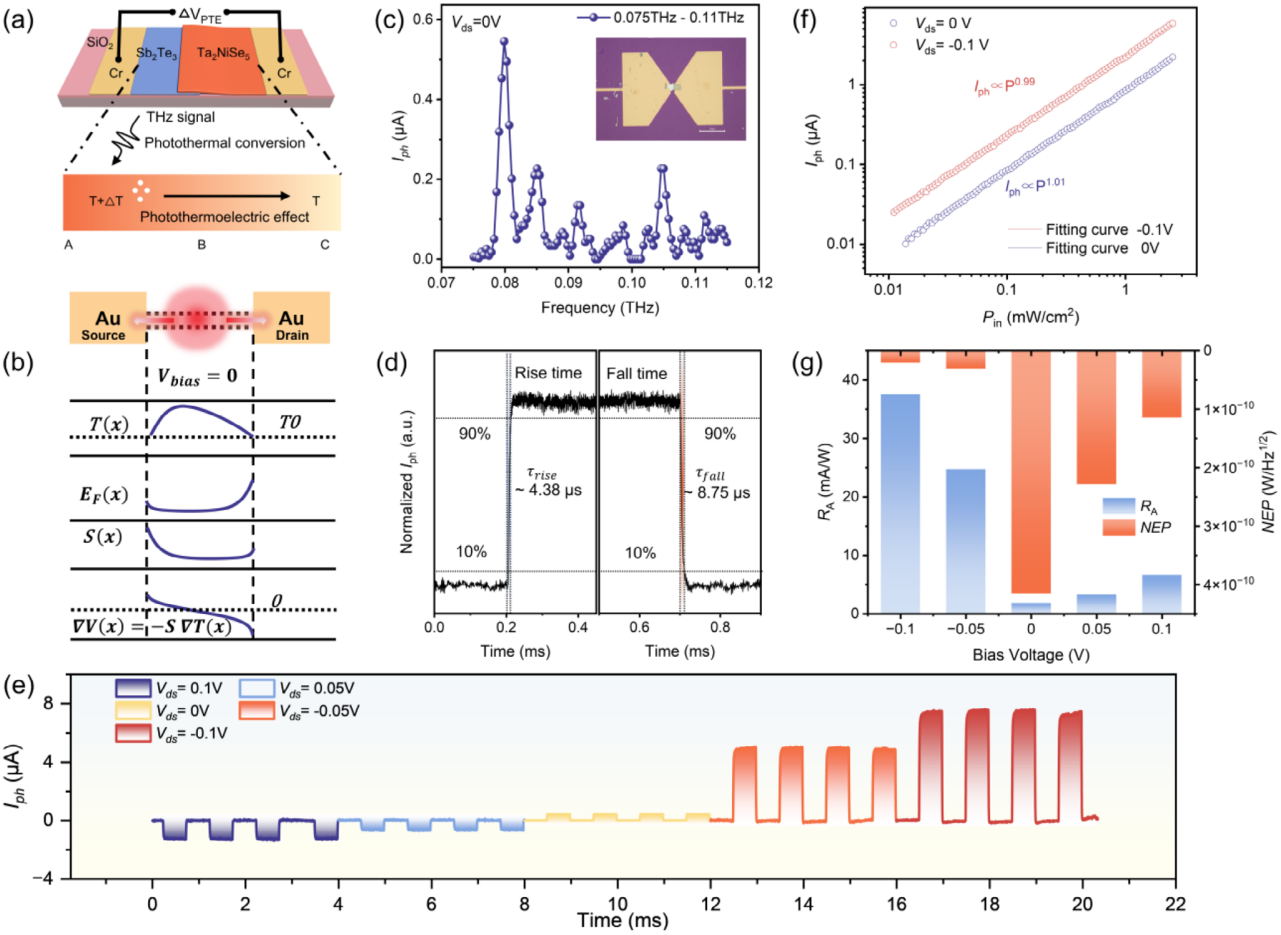
The optical response characteristics of Ta_2_NiSe_5_/Sb_2_Te_3_ van der Waals heterojunction in the THz regime. (a) Schematic illustration of the Ta_2_NiSe_5_/Sb_2_Te_3_ photodetector response mechanism in the THz band. (b) Profiles of the carrier temperature *T*(x), Fermi energy *E*_F_(x), Seebeck coefficient *S*(x), and potential gradient based on heterojunction and butterfly junction electrodes without bias voltage. (c) Photocurrent of the Ta_2_NiSe_5_/Sb_2_Te_3_ photodetector at a frequency from 0.075 to 0.11 THz without any bias. The inset shows the optical image of the Ta_2_NiSe_5_/Sb_2_Te_3_ van der Waals heterojunction photodetector. (d) Rising/falling times for the photodetector under 0.10 THz illumination at zero bias. (e) Pulsed photocurrent responses of Ta_2_NiSe_5_/Sb_2_Te_3_ photodetector under bias voltages of 0.1 V, 0.05 V, 0 V, −0.05 V, and −0.1 V, respectively. (f) Photocurrent versus varying modulated frequency, indicating a 3 dB frequency of 10 kHz. The inset shows the photoresponse waveform of the photodetector under 0.10 THz illumination with different bias voltages (−0.1 V, 0 V, and 0.1 V). (g) The responsivity and room temperature NEP of the Ta_2_NiSe_5_/Sb_2_Te_3_ photodetector under different bias voltages.

Conventional semiconductor photodetection, which relies on direct interband excitation of electron–hole pairs, becomes inefficient in the THz regime due to the extremely low photon energy (a few meV). To overcome this challenge, a bow‐tie antenna with a channel length of 500 nm was integrated into the Ta_2_NiSe_5_/Sb_2_Te_3_ heterojunction (Figure 4a), efficiently concentrating incident THz radiation into a localized oscillating electric field across the gap. High‐resistivity intrinsic Si (ρ = 20 000 Ω·cm) was selected as the substrate to suppress parasitic absorption and scattering losses. A lock‐in technique was employed to measure the THz photoresponse using a microwave‐driven frequency‐multiplier chain (VDI) covering 0.075–0.11 THz (Details in the Methods). The mechanism of the Photo‐thermoelectric (PTE) effect is illustrated in Figure 4a,b. To achieve this, the heterojunction is integrated with a bow‐tie antenna that efficiently couples the free‐space THz radiation and concentrates it as a highly localized oscillating field within the device channel. The field drives strong Joule heating of the charge carriers, creating a non‐equilibrium hot carrier distribution and establishing a local temperature gradient (Δ*T*) across the junction. The key to the PTE effect lies in the engineered asymmetry of the heterojunction itself. Due to the dissimilar electronic structures of Ta_2_NiSe_5_/Sb_2_Te_3_, a sharp spatial gradient in the Seebeck coefficient (∇*S*) exists at the interface where the temperature gradient is maximal. The Seebeck coefficient of Sb_2_Te_3_ is 164 µV·K^−1^ [35], and that of Ta_2_NiSe_5_ is 50 µV·K^−1^ [46]. This co‐localization of thermal and thermoelectric gradients generates a net photovoltage, *V*_PTE_ ∝ ∫∇*S*(*x*) Δ*T*(*x*)dx, allowing the device to function as a highly sensitive, zero‐bias thermal detector. To characterize the THz response, a lock‐in amplifier with a VDI frequency multiplier chain (0.075‐0.11THz) was employed. Figure 4c (bow‐tie antenna geometry, channel length ∼500 nm) shows a prominent photocurrent peak at 0.1 THz under zero bias, confirming resonant coupling between the antenna and THz radiation.

The subwavelength gap of bow‐tie antenna geometry concentrates the THz electric field, enhancing absorption and temperature gradient formation, which are critical for PTE efficiency. Figure 4f shows the photocurrent versus incident light power under different voltages for 0.1 THz. By fitting the relationship between photocurrent and light power with the power law (*I*_ph_ ∝ *P* ^α^, *I*_ph_, *P*, and α are photocurrent, incident light power, and ideal factor, respectively), the α is measured to be 0.99 and 1.01 for −0.1 V and 0 V, which indicates a large dynamic range of Ta_2_NiSe_5_/Sb_2_Te_3_ heterojunction photodetector. A single pulse with a modulation frequency of 1 kHz is recorded by an oscilloscope, as shown in Figure 4e. The rise time and fall time are 4.38 µs and 8.75 µs, respectively. The fast dynamics originate from the high thermal conductivity of Sb_2_Te_3_ and the efficient heat dissipation of Ta_2_NiSe_5_, enabling rapid thermal equilibrium, which is beneficial for real‐time imaging and real‐time communication.

The responsivity (R) and noise‐equivalent power (NEP) were further quantified. Taking the antenna‐coupled effective detection area as the diffraction‐limited spot size, the peak responsivity reaches 29.1 mA·W^−1^, while the minimum NEP achieves ≈ 26.1 pW·Hz^−1/2^ (Figure 4f). At the operating frequency of 1 kHz, 1/*f* noise is negligible, and shot noise is strongly suppressed under zero bias, therby rendering thermal noise the dominant contribution. Together, these metrics benchmark the Ta_2_NiSe_5_/Sb_2_Te_3_ heterojunction as a competitive candidate for sensitive, bias‐free THz detection.

**FIGURE 5:**
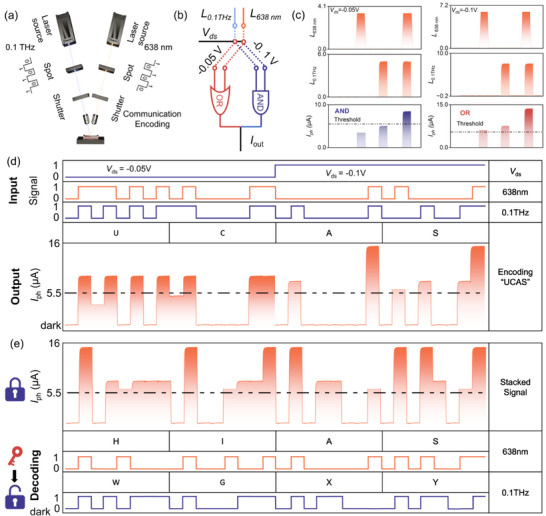
Photoelectric logic gate based on Ta_2_NiSe_5_/Sb_2_Te_3_ heterojunction ultrabroadband photodetector. (a) The schematic diagram of the logic gates using the Ta_2_NiSe_5_/Sb_2_Te_3_ photodetector. (b) The proposed concept diagram of logic gates. The input signals are 638 nm and 0.1 THz light sources, while the source‐drain voltage of −0.05 V and −0.1 V modulate the channel carrier concentration, thereby controlling the output current and switching the device to switch between “AND” and “OR” logic functions. (c) Photocurrent level of the “AND” and “OR” logic gates with a threshold of 6.5 µA. (d) Input and output signals are used to transmit the ASCII code “UCAS” in ultra‐wide spectrum communication systems. (e) “HIAS” emitted by a 638 nm laser is used as the key to decode the superimposed signal to recover the real signal “WGXY” emitted by a 0.1 THz light source.

Table 1 compares the performance of our device with state‐of‐the‐art ultrabroadband photodetectors based on 2D materials. Distinct from devices limited to either VIS−NIR or THz ranges, the Ta_2_NiSe_5_/Sb_2_Te_3_ heterojunction device uniquely bridges both regimes within a single platform, offering fast response, low NEP, and broadband operability under ambient conditions. The broadband responsivity of the Ta_2_NiSe_5_/Sb_2_Te_3_ heterojunction, spanning from the VIS to the THz regime, enables its integration into multifunctional optoelectronic circuits capable of simultaneously receiving fiber‐optic and wireless signals. Figure 5a shows the configuration of the photodetector system, showing a multi‐wavelength light source incident on the Ta_2_NiSe_5_/Sb_2_Te_3_ heterojunction, realizing the basic logic functions of “AND” and “OR” by varying the source‐drain voltage and inputting the dual‐channel signals, and further applying the device to ASCII message transmission. The structure of the proposed logic gate is shown in Figure 5b. Two optical inputs—638 nm (channel 1) and 0.1 THz (channel 2) are encoded as binary logic levels of the gate type, while bias modulation provides electrical programmability. Specifically, at *V*_ds_ = −0.05 V, the heterojunction operates as an “AND” gate, with photocurrent exceeding the threshold only when both optical inputs are simultaneously present. Conversely, at *V*_ds_ = −0.1 V, the device functions as an “OR” gate, producing an output above the threshold under illumination from either channel individually or jointly (Figure 5c).

**TABLE 1:** Performance comparison of the Ta_2_NiSe_5_/Sb_2_Te_3_ heterojunction photodetector with other 2D photodetectors at room temperature.

Type	Response range	λ (nm)	Bias (V)	R (A/W)	*D* ^*^(Jones)	τ	Refs.
Ta_2_NiSe_5_	520 nm–4600 nm	4600	1	0.86	8.75 × 10^8^	24/26 ms	[47]
Sb_2_Te_3_	635 nm–0.34 THz	635	0.2	1.1	—	94/98 µs	[28]
WSe_2_/Ta_2_NiSe_5_/WSe_2_	400 nm—1550 nm	635	0	0.436	1 × 10^12^	420/640 µs	[48]
Ta_2_NiSe_5_/MoS_2_	532/1064 nm	1064	3	0.7	2.4 × 10^9^	7.4/31.1 s	[21]
Ta_2_NiSe_5_/GaSe	520/1550 nm	1550	−3	0.15	1.08 × 10^9^	370/540 ms	[49]
Ta_2_NiSe_5_/WSe_2_	638/1550 nm	1550	0	0.82 × 10^−9^	90	278/283 µs	[50]
Ta_2_NiSe_5_/ a‐In_2_Se_3_	405 nm–1550 nm	520	0.5	12	3 × 10^13^	25/423 µs	[51]
Ta_2_NiSe_5_/Graphene	638 nm–0.3 THz	638	1	4.8 × 10^−3^	1.9 × 10^6^	7.4/6.3 µs (0.12 THz)	[52]
Ta_2_NiSe_5_/p‐GaN	355 nm	355	0	0.61	2.0 × 10^10^ (5 V)	0.6/0.67 ms	[53]
Ta_2_NiSe_5_/MoSe_2_	783 nm	783	−1	0.5	1.14 × 10^11^	169/180 µs	[54]
Ta_2_NiSe_5_/WSe_2_	532 nm	532	3	12.5 × 10^−3^	5.1 × 10^9^	1.7/2 ms	[55]
Sb_2_Te_3_/Bi_2_Te_2_Se	600 nm–1100 nm	1000	1	112	3.44 × 10^12^	—	[56]
Sb_2_Te_3_/WS_2_	476 nm–2200 nm	1310	1	0.429	1.89 × 10^9^	12/24 ms (1550 nm)	[39]
Sb_2_Te_3_/Bi_2_Se_3_	450 nm–1550 nm	520	0.5	0.05	—	300 /300 µs	[42]
Sb_2_Te_3_/MoS_2_	500 nm–900 nm	500	0.2	0.12	—	0.197/0.138 s	[57]
Sb_2_Te_3_/MoSe_2_	532 nm–1550 nm	532	1	35	6.5 × 10^11^	110/230 µs	[58]
Sb_2_Te_3_/p++‐Si	500 nm	500	10	0.45	1.38 × 10^10^	15.15/14.03 s	[59]
Sb_2_Te_3_/WSe_2_	532 nm–1550 nm	532	0.2	0.41	3.6 × 10^10^	81/160 µs	[60]
Ta_2_NiSe_5_/Sb_2_Te_3_	638 nm–0.1 THz	940	0.1	0.19	1.33 × 10^8^	11.8/5.9 ms	This work
		0.1 THz	−0.1	0.014	—	4.38/8.75 µs	

The complementary polarity of the VIS and THz‐induced photocurrents further facilitates dual‐channel discrimination and reduces crosstalk. As shown in Figure 5c, for the light response of 638 nm and 0.1 THz, the turn‐on is “1” and the turn‐off is “0”. When the photocurrent exceeds the threshold 6.5 µA, the output signal is defined as “1”. Switching between the “AND” and “OR” logic gate functions can be easily realized by electrically modulating the photodetector heterojunction carrier concentration. For the “AND” gate, the *V*_ds_ is held at −0.05 V, and only when two light beams are irradiated simultaneously does the time current exceed the threshold, and the output signal is defined as “1”. In the “OR” gate, the *V*_ds_ is tuned to −0.1 V, and the output signal is “1” regardless of individual or simultaneous irradiation.

To address the limitations of single‐channel communication, including susceptibility to interference and poor confidentiality, we expect to utilize the Ta_2_NiSe_5_/Sb_2_Te_3_ detector with a broadband response as a dual‐channel or even multichannel signal‐receiving unit for encrypted and antijamming information transmission. As shown in Figure 5d, the specially modulated 638 nm and 0.1 THz light sources act as transmitters to send the “UCAS” message, with the *V*_ds_ simultaneously tuned to modify the logic functions. The detector subsequently retrieves the ASCII code “0 101 0101 0 100 0011 0 100 0001 0 101 0011”, which corresponds to the characters “U”, “C”, “A”, and “S”, respectively. Furthermore, one of the beams can be employed as a key to decode the mixed information detected by the Ta_2_NiSe_5_/Sb_2_Te_3_ detector. As illustrated in Figure 5e, for the stacked signal illuminated by two light sources, if the ASCII code “0 100 1000 0 100 1001 0 100 0001 0 101 0011” of the character “HIAS” emitted by the 638 nm light source is known, the ASCII code “0 101 0111 0 100 0111 0 101 1000 0 101 1001” emitted by the 0.1 THz light source can be decoded to obtain the character “WGXY”, thereby removing interference signals from the stacked signal and restoring the original information. The design of ultrabroadband spectrum photodetectors based on Ta_2_NiSe_5_/Sb_2_Te_3_ heterojunction provides a potential solution for low‐cost, integrated, and simple development of encrypted communication systems.

## 3 Conclusion

In summary, we have demonstrated that the Ta_2_NiSe_5_/Sb_2_Te_3_ van der Waals heterojunction enables high‐performance ultrabroadband photodetection spanning the VIS to THz regimes at room temperature. Distinct physical mechanisms—photoconductive processes in the VIS–NIR and photothermoelectric conversion in the THz—cooperatively yield responsivities up to 188 mA·W^−1^ (940 nm) and 29 mA·W^−1^ (0.1 THz), with a minimum NEP of 26 pW·Hz^−1/2^. The device further delivers µs‐scale THz response times, ensuring operational stability and reproducibility. Beyond detection, reconfigurable optoelectronic logic gates (“AND”/“OR”) and dual‐channel ASCII transmission were realized, underscoring its potential for secure and anti‐jamming communication. These results establish the Ta_2_NiSe_5_/Sb_2_Te_3_ platform as a scalable route toward integrated, room‐temperature broadband optoelectronics, offering new opportunities for real‐time imaging, high‐capacity communications, and multifunctional photonic–electronic systems.

## 4 Methods

### 4.1 Sample and Device Preparation

The bulk Ta_2_NiSe_5_ and Sb_2_Te_3_ were purchased from the Shanghai Onway Technology Co., Ltd. The 2D Ta_2_NiSe_5_ and Sb_2_Te_3_ nanoflakes were obtained by using the mechanical stripping method to strip them from the bulk crystal. The 2D Sb_2_Te_3_ nanoflake was first transferred onto a low‐resistance silicon substrate with a 300 nm insulating layer of SiO_2_ via poly(dimethyl siloxane) (PDMS), and then the 2D Ta_2_NiSe_5_ nanoflake was stacked onto the Sb_2_Te_3_. The bottom electrodes were patterned using a Semi‐Automated Mask Aligner (MA/BA6 Gen4), and then metal contacts (Cr/Au = 5 nm/50 nm) were deposited by high vacuum evaporation technology (Ei‐5Z), and then they went through a lift‐off process.

### 4.2 Material Characterization

The thickness of the Ta_2_NiSe_5_ and Sb_2_Te_3_ nanoflakes was measured using an AFM (Dimension ICON). The micro‐Raman system (Renishaw in Via) with a 532 nm laser was used to obtain the Raman spectrum. The PL spectra were characterized by the confocal microscope (Horiba Scientific LabRAM HR Evolution) and a sequential wavelength 532 nm laser.

### 4.3 Band Calculation

First‐principles density functional theory (DFT) calculations were performed using the Vienna ab initio simulation package (VASP) [61, 62]. The projector augmented wave (PAW) method [63] was employed in combination with the generalized gradient approximation (GGA) in the Perdew‐Burke‐Ernzerhof (PBE) form [64]. A kinetic energy cutoff of 400 eV was used to expand the wave functions into a plane‐wave basis, and the energy convergence criterion was set to 10^−7^ eV. The Brillouin zone was sampled using a Γ‐centered Monkhorst–Pack k‐mesh with a target k‐point spacing of 0.02, Å^−1^ [65]. To eliminate interlayer interactions, a sufficiently large vacuum spacing of 20 Å was introduced along the out‐of‐plane direction. The structural relaxations were performed until the Hellmann‐Feynman forces acting on each atom were reduced to less than 0.01 eV·Å^−1^.

### 4.4 Electrical and Photoresponse Measurements

*VIS–NIR*. The electrical transport behavior and optoelectronic performance were all characterized at room temperature and in ambient air. A highly sensitive dual‐channel digital source meter (Keithley 6482) was used for applying the bias and measuring the current and photovoltage at the same time. The Ta_2_NiSe_5_/Sb_2_Te_3_ heterojunction photodetector was mounted on a sophisticated sample holder to increase stability and precision during measurement. For the basic photoelectric test and performance calculation, the incident light path of the laser was a uniform spot with a spot diameter of 27 µm (520–1650 nm), focused by using a 50X objective lens. The effective light power density (*P*_eff_) was calculated using *P*_eff_ = *P*_in_ × *A*_eff_ = *P*_in_ × *A*_device_/*A*_spot_, where the effective photoresponse area of the heterojunction *A*_device_ = 8 µm × 11 µm = 88 µm^2^. The photocurrent mapping was performed by scanning the pressure point console in horizontal and vertical directions under the irradiation of a focused modulated laser, and the photocurrent signal was obtained using a lock‐in amplifier (MStarter 200). Shot noise can be evaluated by *i*_s_^2^ = 2*eI*_d_Δ*B* [66], where Δ*B* is the bandwidth, At *V*_ds_ = 0.1 V, *I*_d_ = 114.4 µA, and Δ*B* = 1 Hz, *i*_s_^2^ = 3.66 × 10^−23^ A^2^ ·Hz^−1^. The Johnson noise level can be expressed as *i*_t_^2^ = 4*k*_B_ *TB*/*R*_0_, where *k*_B_ is the Boltzmann constant, and *R*_0_ is the device resistance. The Johnson noise *i*_t_^2^ = 1.89 × 10^−23^ A^2^ ·Hz^−1^ is obtained at Δ*B* = 1 Hz, and *R*_0_ = 874.1 Ω for the 0.1 V bias, and *T* = 300 K, *k*_B_ = 1.38 × 10^−23^ J·K^−1^. The EQE and NEP can be calculated from the following equation: EQE = *hcR*_A_/eλ, NEP = *i*_n_/*R*_A_ [67], where *h* is 6.626 × 10^−34^, *c* is 3 × 10^8^ m·s^−1^, *e* is 1.6 × 10^−19^ C and *i*_n_ = (*i*_t_^2^ + *i*_s_^2^)^1/2^ = (1.89 × 10^−23^ + 3.66×10^−23^)^1/2^ A·Hz^−1/2^ = 74.5 pA·Hz^−1/2^. The responsivity (R) is defined as the ratio of the photocurrent or photovoltage of the photodetector to the incident light power [68]: *R*_A_ = *I*_ph_/*P*_eff_ = (*I*_ds_−*I*_dark_)/(*P*_in_·*A*_eff_), *I*_ph_ is the photocurrent, *I*_dark_ is the dark current, *P*_eff_ is the effective incident light intensity, *P*_in_ is the incident light power, and *A*_eff_ is the effective illumination area. *D* ^*^ can measure the ability of photodetectors to detect weak optical signals [67]: *D* ^*^ = (*A*·Δ*f*)^1/2^/NEP = (*A*·Δ*f*)^1/2^ *R*_A_/*i*_n_, where *A* is the effective area of the device, Δ*f* = 1 Hz, and the noise equivalent power can be expressed as NEP = *i*_n_/*R*_A_.

Furthermore, the Keithley 4200A‐SCS semiconductor parameter analyzer was adopted to measure the voltage noise spectra. The noise equivalent power (NEP) is calculated as the ratio of noise voltage density to responsivity: NEP = *v*_n_ /*R*_V_. 1/*f* noise is negligible since our device operates at 1 kHz, and operating in zero bias condition, the contribution of shot noise to the entire noise spectrum can also be ignored [69]. The contribution of thermal noise *v*_t_ to the noise spectral density at room temperature through $v_{t}=\sqrt{4k_{B}TR}$ is ≈ 21.75 nV·Hz^−1/2^. As shown in Figure 4g, as an important performance index, the maximum value of *R*_A_ is 29.1 mA·W^−1^, and the minimum value of NEP is about 26.1 pWHz^−1/2^ at ambient temperature.

## Conflicts of Interest

The authors declare no conflicts of interest.

## Supporting information




**Supporting File**: advs73795‐sup‐0001‐SuppMat.docx.

## Data Availability

The data that support the findings of this study are available from the corresponding author upon reasonable request.
