# Supplementary material for: Interfacial Synergy in a Band‐Aligned Low‐Dimensional Heterojunction Toward Broadband Photodetection
Source: Adv Sci (Weinh). 2026 Feb 26;13(20):e20919. doi: 10.1002/advs.202520919 (PMC13067850; doi:10.1002/advs.202520919)
Supplement: Supplementary file 1 — Supporting File: advs73795‐sup‐0001‐SuppMat.docx. [file ADVS-13-e20919-s001.docx]

Supporting Information

Interfacial Synergy in a Band-Aligned Low-Dimensional Heterojunction toward Broadband Photodetection

*Yuanfeng Wen^1§^, Kening Xiao^1§^, Yao Yang^2§^, Mengjie Jiang^1^, Libo Zhang^1^*, Shicong Hou^4^, Hang Ma^1^, Yunduo Zhang^1^, Wenqi Mo^1^, Yiran Tan^1^, Bolang Peng^1^, Jinqiu Huang^1^, Xueyuan Wei^1^, Bangjian Zhao^1*^, Jiale He^1*^, Qing Li^1^, Songyuan Ding^1^, Yi Zhou^1^, Guanhai Li^1,3*^, Xiaoshuang Chen^1,3,5^*

^1^College of Physics and Optoelectronic Engineering, Hangzhou Institute for Advanced Study, University of Chinese Academy of Sciences, No. 1, Sub-Lane Xiangshan, Xihu District, Hangzhou 310024, China.

^2^College of Physics and Optoelectronic Engineering, Shenzhen University, Shenzhen 518060, China.

^3^State Key Laboratory of Infrared Physics, Shanghai Institute of Technical Physics, Chinese Academy of Sciences, 500 Yu-Tian Road, Shanghai 200083, China.

^4^Shanghai Key Lab of Modern Optical System, University of Shanghai for Science and Technology, 516 Jungong Road, Shanghai 200093, China.

^5^Suzhou Laboratory, Suzhou, Jiangsu, 215000, P. R. China.

**Main contents**

1. KPFM measurement of the Ta_2_NiSe_5_/Sb_2_Te_3_ heterojunction
2. Band structures of monolayer Ta_2_NiSe_5_ calculated by DFT.
3. Band structures of monolayer Sb_2_Te_3_ calculated by DFT.
4. Photocurrent mapping diagram of Ta_2_NiSe_5_/Sb_2_Te_3_ heterojunction photodetector
5. Photocurrent switching characteristics of Ta_2_NiSe_5_/Sb_2_Te_3_ heterojunction photodetector with various light power.
6. The photocurrent switching characteristics of Ta_2_NiSe_5_/Sb_2_Te_3_ heterojunction photodetectors under different optical powers at bias voltage of 0 V.
7. The responsivity of Ta_2_NiSe_5_/Sb_2_Te_3_ photodetector varies with the incident light power.
8. *D*^*^ and EQE of Ta_2_NiSe_5_/Sb_2_Te_3_ heterojunction devices
9. The *V*_ds_-*I*_ds_ diagram of Ta_2_NiSe_5_/Sb_2_Te_3_ heterojunction photodetector under 0.1THz illumination.
10. Photocurrent of the Ta_2_NiSe_5_/Sb_2_Te_3_ photodetector at THz frequency.
11. Profiles across the carrier *T*(*x*), *E*_F_(*x*), *S*(*x*), and potential gradient based on heterojunction and butterfly junction electrodes at different bias voltage.
12. Photocurrent versus varying modulated frequency in the THz region.

**1. KPFM measurement of the Ta_2_NiSe_5_/Sb_2_Te_3_ heterojunction**

KPFM measurement was done to investigate band alignment of our Ta_2_NiSe_5_/Sb_2_Te_3_ heterojunction. Figure S1 shows a KPFM image of a typical Ta_2_NiSe_5_/Sb_2_Te_3_ heterojunction with contact potential difference Δ*V*_CPD_ between KPFM tip and the measured surface. Before measurement, the KPFM tip was calibrated by Au surface with work function around 5.1 eV, thus the work function of the tip was determined by *Φ*_tip_ = *Φ*_Au_ + Δ*V*_CPD_ = *Φ*_Au_ + e·Δ*CPD*_(tip-Au)_ =5.1 eV - 0.384 eV = 4.716 eV. Δ*CPD*_(tip-Au)_ is the difference of CPD between tip and Au which is equal to the difference of surface potentials between tip and Au. Therefore, the work functions of Sb_2_Te_3_ and Ta_2_NiSe_5_ estimated by *Φ*_sample_ = *Φ*_tip_ − Δ*V*_CPD_ = *Φ*_tip_ − e·Δ*CPD*_(tip-sample)_ can be derived from the KPFM measurement(Δ*CPD*_(tip-Sb2Te3)_ = -0.251 eV, Δ*CPD*_(tip-Ta2NiSe5)_ = -0.366 eV), which is ~ 4.967 eV and ~ 5.082 eV, respectively.


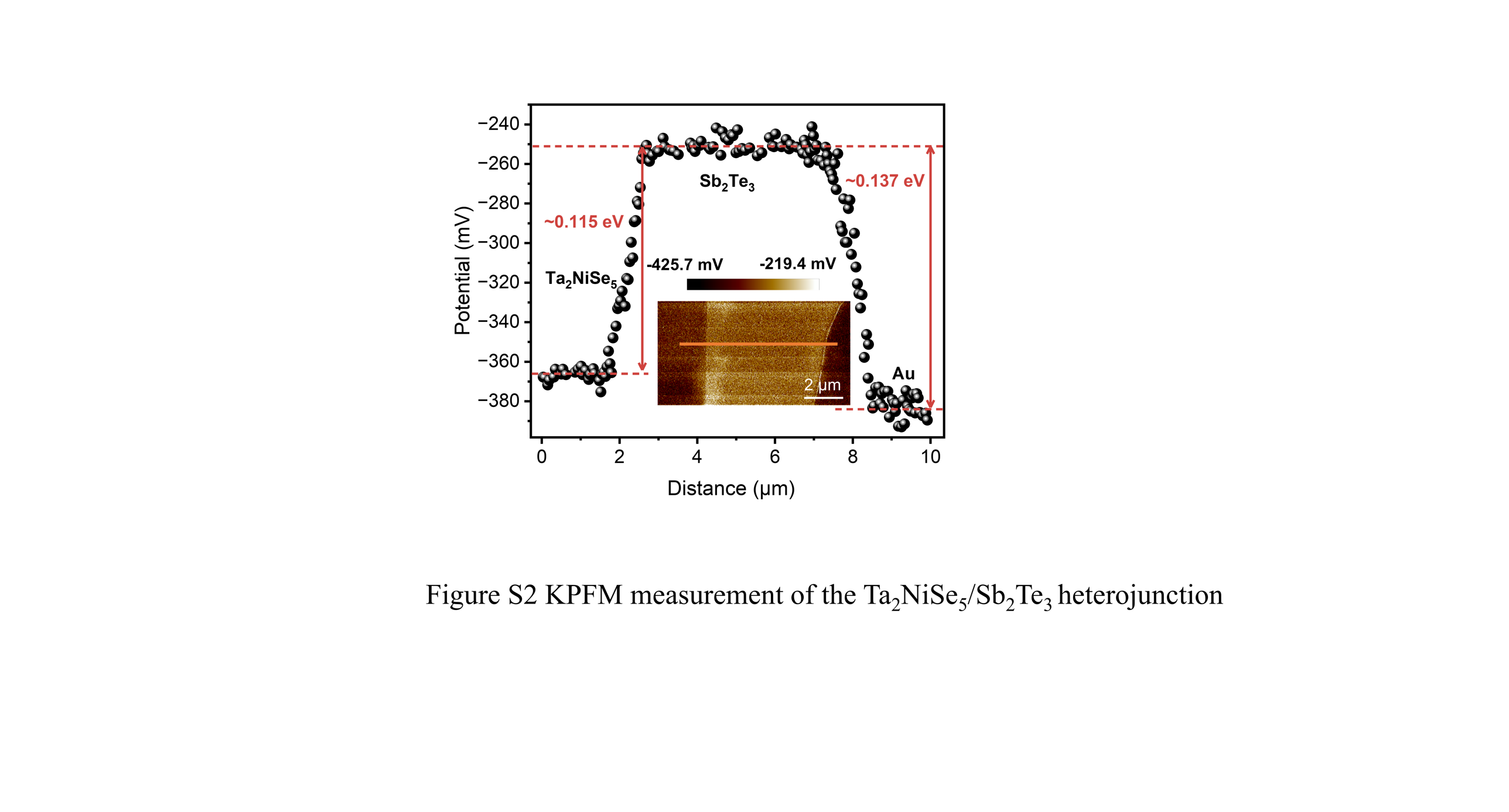


**Figure S1 KPFM measurement of the Ta_2_NiSe_5_/Sb_2_Te_3_ heterojunction.** KPFM measurement of the potential difference between Ta_2_NiSe_5_/Sb_2_Te_3_ heterojunction and Au electrode along the orange line. The inset image is the KPFM measurement mapping of the Ta_2_NiSe_5_/Sb_2_Te_3_ heterojunction photodetector.

**2. Band structures of monolayer Ta_2_NiSe_5_ calculated by DFT.**

The Figure S2a-d shows the band structures of Ta_2_NiSe_5_ with 1 layer, 2 layers, 3 layers and 4 layers calculated by DFT. In the energy band structure of monolayer Ta_2_NiSe_5_ in Figure S2a, the dispersion of conduction band and valence band is significant, and the Fermi level is located near the intersection of conduction band and valence band, showing metal or semimetal behavior. For the 2-layer structure (Figure S2b), the band structure shows enhanced inter-layer hybridization, which leads to a change in band dispersion. Under the three-layer structure (Figure S2c), the band structure further changes, indicating that the interlayer coupling effect is more significant. Compared with the single layer, the band dispersion is reduced, and more energy states appear near the Fermi level. In the 4-layer structure (Figure S2d), the band structure has changed significantly compared with the thinner layer, and the band structure in some regions has become more flat. It shows that the interlayer interaction is stronger and the material exhibits stronger semiconductor or insulation characteristics.

**
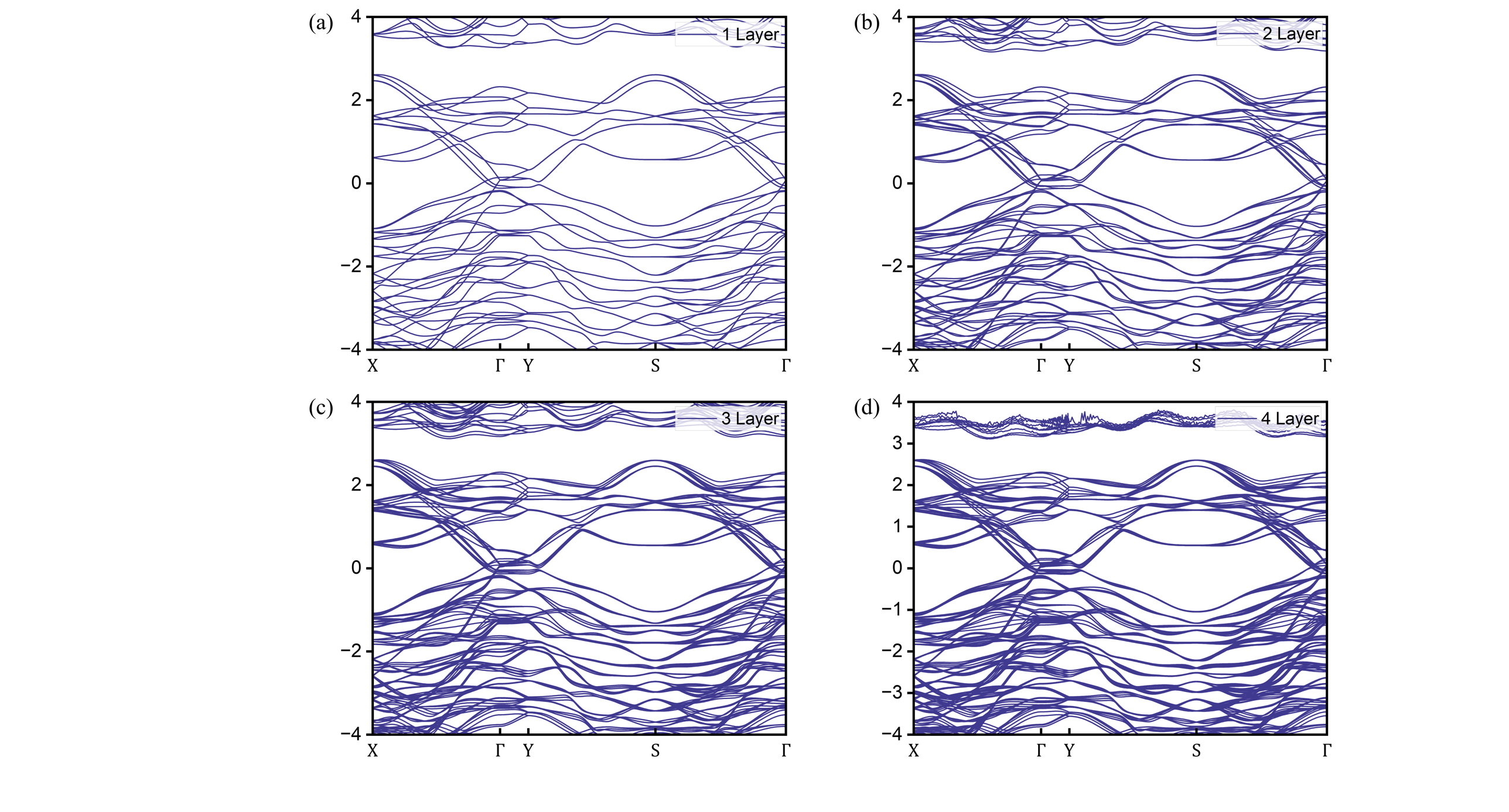
**

**Figure S2 Band structures of monolayer Ta_2_NiSe_5_ calculated by DFT.** a) The band structure of 1-layer Ta_2_NiSe_5_ was calculated by DFT. b) The band structure of 2-layer Ta_2_NiSe_5_ was calculated by DFT. c) The band structure of 3-layer Ta_2_NiSe_5_ was calculated by DFT. d) The band structure of 4-layer Ta_2_NiSe_5_ was calculated by DFT.

**3. Band structures of monolayer Sb_2_Te_3_ calculated by DFT.**

The Figure S3a-d shows the band structures of Sb_2_Te_3_ with 1 layer, 2 layers, 3 layers and 4 layers calculated by DFT. With the increase of the number of Sb_2_Te_3_ layers, the band structure of the material has experienced from a more complex single-layer crossover state to a gradually stable semiconductor characteristic. The energy band difference between different layers shows that the increase of the number of layers leads to the enhancement of the interlayer interaction, and gradually inhibits the performance of the surface state, and finally tends to a more stable electronic state.

**
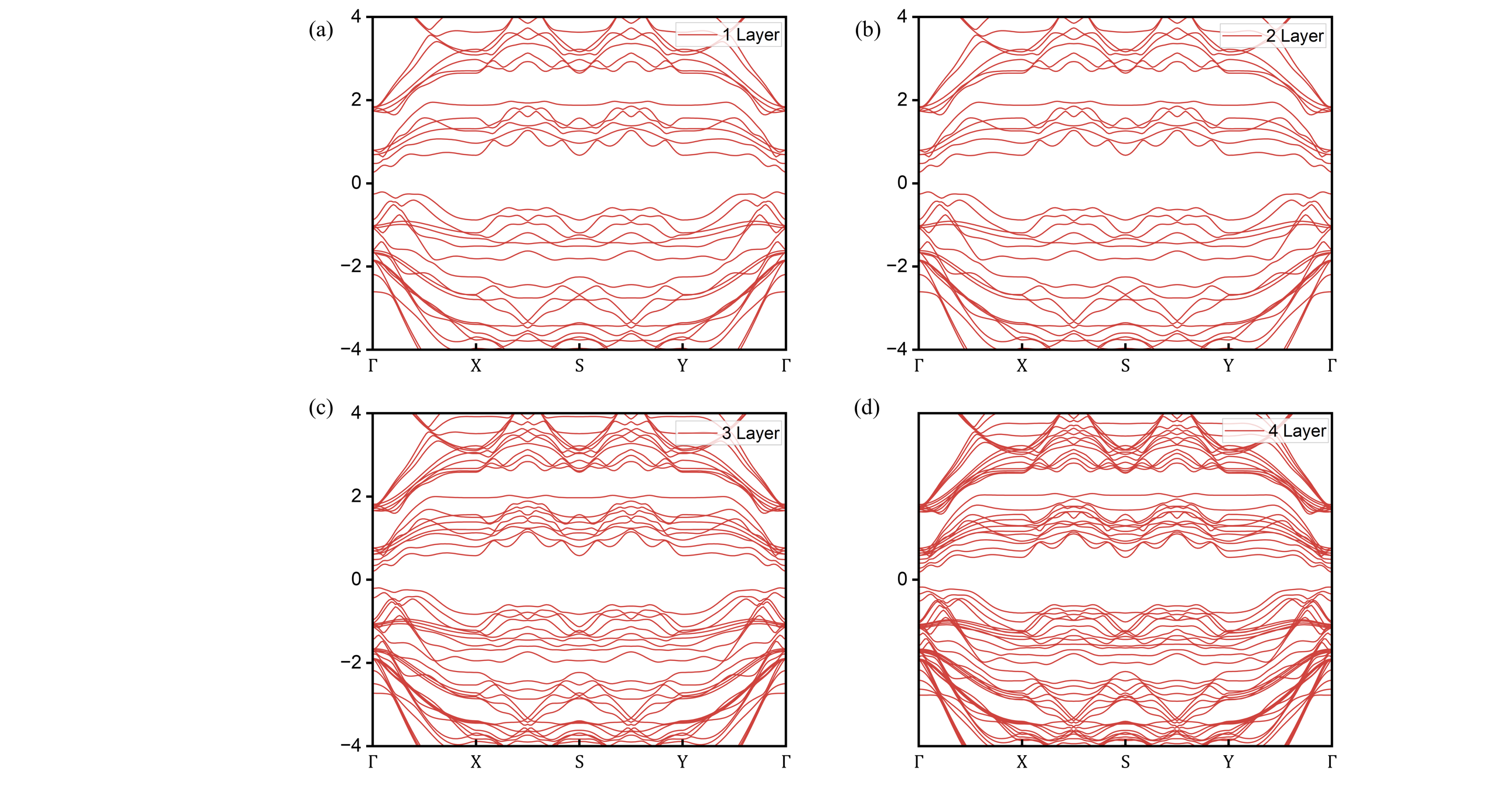
**

**Figure S3 Band structures of monolayer Sb_2_Te_3_ calculated by DFT.**

a) The band structure of 1-layer Sb_2_Te_3_ was calculated by DFT. b) The band structure of 2-layer Sb_2_Te_3_ was calculated by DFT. c) The band structure of 3-layer Sb_2_Te_3_ was calculated by DFT. d) The band structure of 4-layer Sb_2_Te_3_ was calculated by DFT.

**4. Photocurrent mapping diagram of Ta_2_NiSe_5_/Sb_2_Te_3_ heterojunction photodetector**

Figure S4a-c gives the Photocurrent mapping diagram of Ta_2_NiSe_5_/Sb_2_Te_3_ heterojunction in the communication band of 1550 nm with Plight = 89.4 μW. As an important band in the communication field, the response of the device in this band is crucial for its successful application in on-chip integrated communication. Figure S4a.b shows that the photoresponse of Ta_2_NiSe_5_/Sb_2_Te_3_ heterojunction at 1550 nm under bias is mainly concentrated in the heterojunction region. Figure S4b shows the response at zero bias, indicating that the device can be applied to self-driven on-chip integration. The photocurrent response at 1550 nm (photon energy ~0.8 eV) originates from the synergistic effect of the Ta_2_NiSe_5_/Sb_2_Te_3_ Type-II band alignment and regulated photogenerated carrier dynamics. The 115 meV built-in potential induced by the Type-II alignment creates an interfacial electric field that efficiently separates photogenerated electron-hole pairs—confining electrons to Ta_2_NiSe_5_ (higher electron affinity) and holes to Sb_2_Te_3_ (higher work function)—suppressing recombination. Ta_2_NiSe_5_’s narrow bulk bandgap (0.33 eV) enables strong absorption of 1550 nm photons, while its high carrier mobility (>556.5 cm²·V⁻¹·s⁻¹) accelerates carrier transport across the heterojunction.


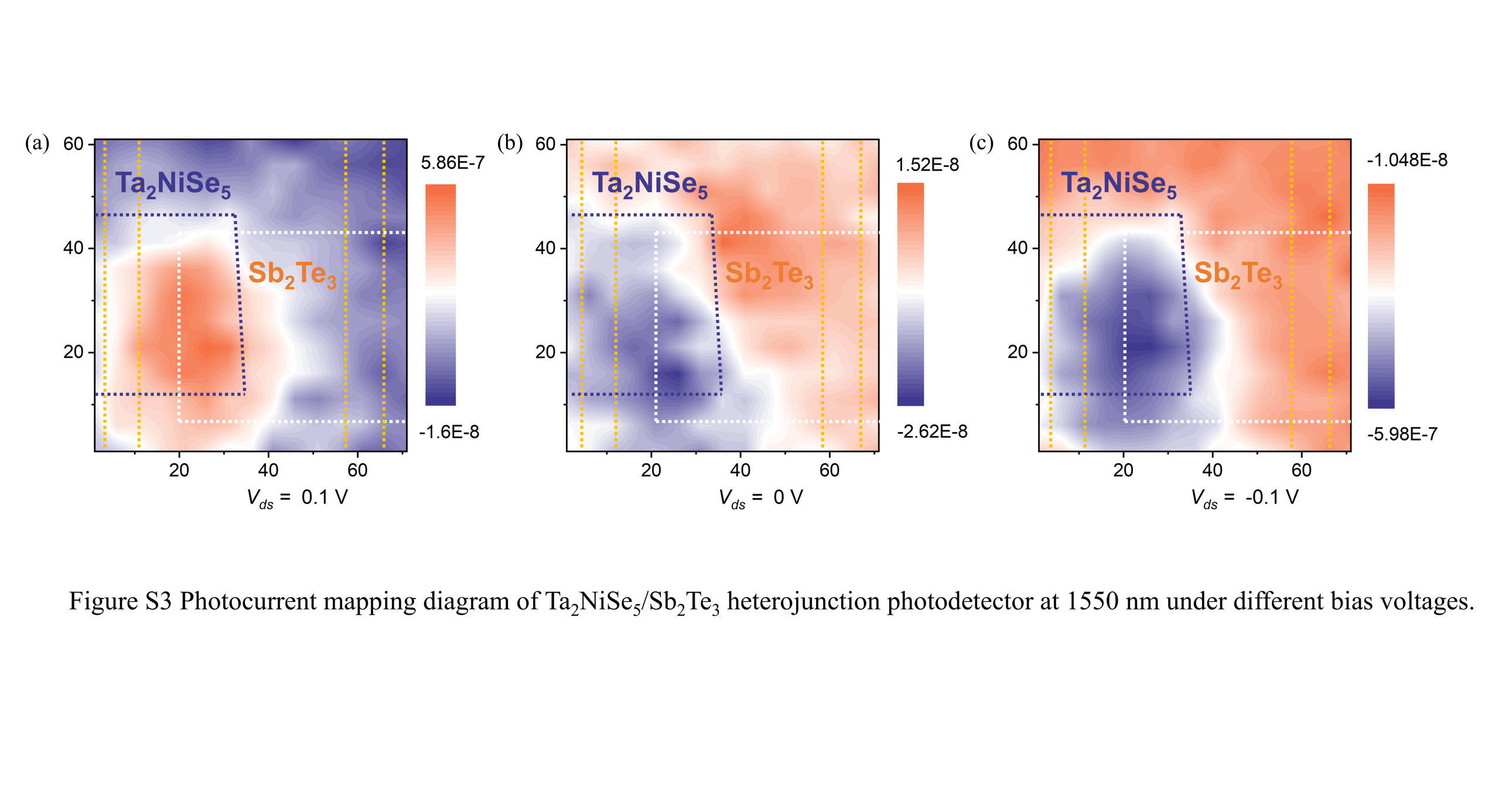


**Figure S4 Photocurrent mapping diagram of Ta_2_NiSe_5_/Sb_2_Te_3_ heterojunction photodetector at 1550 nm under different bias voltages.** a) Photocurrent mapping images correspondingly measured at 0.1 bias under 1550 nm laser illumination. b) Photocurrent mapping images correspondingly measured at 0 bias under 638 nm laser illumination. c) Photocurrent mapping images correspondingly measured at −0.1 V bias under 638 nm laser illumination.

**5. Photocurrent switching characteristics of Ta_2_NiSe_5_/Sb_2_Te_3_ heterojunction photodetector with various light power.**

Figures S5a-d demonstrate that the heterojunction photodetector exhibits stable and rapid photocurrent switching characteristics when exposed to 638, 940, 1064, and 1650 nm lasers. It can be observed from the figure that the device has a high dark current. The high dark current mainly stems from two factors: (i) Interfacial defects (e.g., dangling bonds) in Ta_2_NiSe_5_/Sb_2_Te_3_ heterojunctions induce Shockley-Read-Hall recombination, promoting carrier leakage. (ii) The narrow bandgap of Sb_2_Te_3_ and weak Schottky barrier at metal-semiconductor interfaces facilitate thermionic emission at room temperature.





**Figure S5 The photocurrent switching characteristics of Ta_2_NiSe_5_/Sb_2_Te_3_ heterojunction photodetectors under 0.1V bias voltages in 638 nm to 1550 nm.** a-d) The temporal photoresponse of Ta_2_NiSe_5_/Sb_2_Te_3_ heterojunction photodetector from 638 nm to 1550 nm under various incident light powers. All measurements were carried out in ambient air tested by our source meter (Keithley 6482) at a modulation frequency of 0.5 Hz.

**6. The photocurrent switching characteristics of Ta_2_NiSe_5_/Sb_2_Te_3_ heterojunction photodetectors under different optical powers at bias voltage of 0 V.**

In order to study the photocurrent performance of the Ta_2_NiSe_5_/Sb_2_Te_3_ heterojunction photodetector under self-driving, we measured the photocurrent response of the device at 0V. As shown in Figure R1, the device exhibits a maximum photocurrent response of --1.4 μA at 940 nm, 0.25 μA at 638 nm and 0.021 μA at 1550 nm, corresponding to Figure 2d in the paper and Figure S4a in the supplementary material. The photocurrent at this time originates from the 115 meV built-in potential at the Ta_2_NiSe_5_/Sb_2_Te_3_ interface. The electron-hole pairs generated by photon excitation are spatially separated under the action of the built-in electric field (electrons to Ta_2_NiSe_5_, holes to Sb_2_Te_3_), and the photovoltaic photocurrent can be formed without external bias, and the photocurrent is limited to the heterojunction interface. This indicates that the prepared Ta_2_NiSe_5_/Sb_2_Te_3_ heterojunction photodetector has good self-driving ability, which can provide an excellent solution for future self-driving ultra-wide spectrum detection.


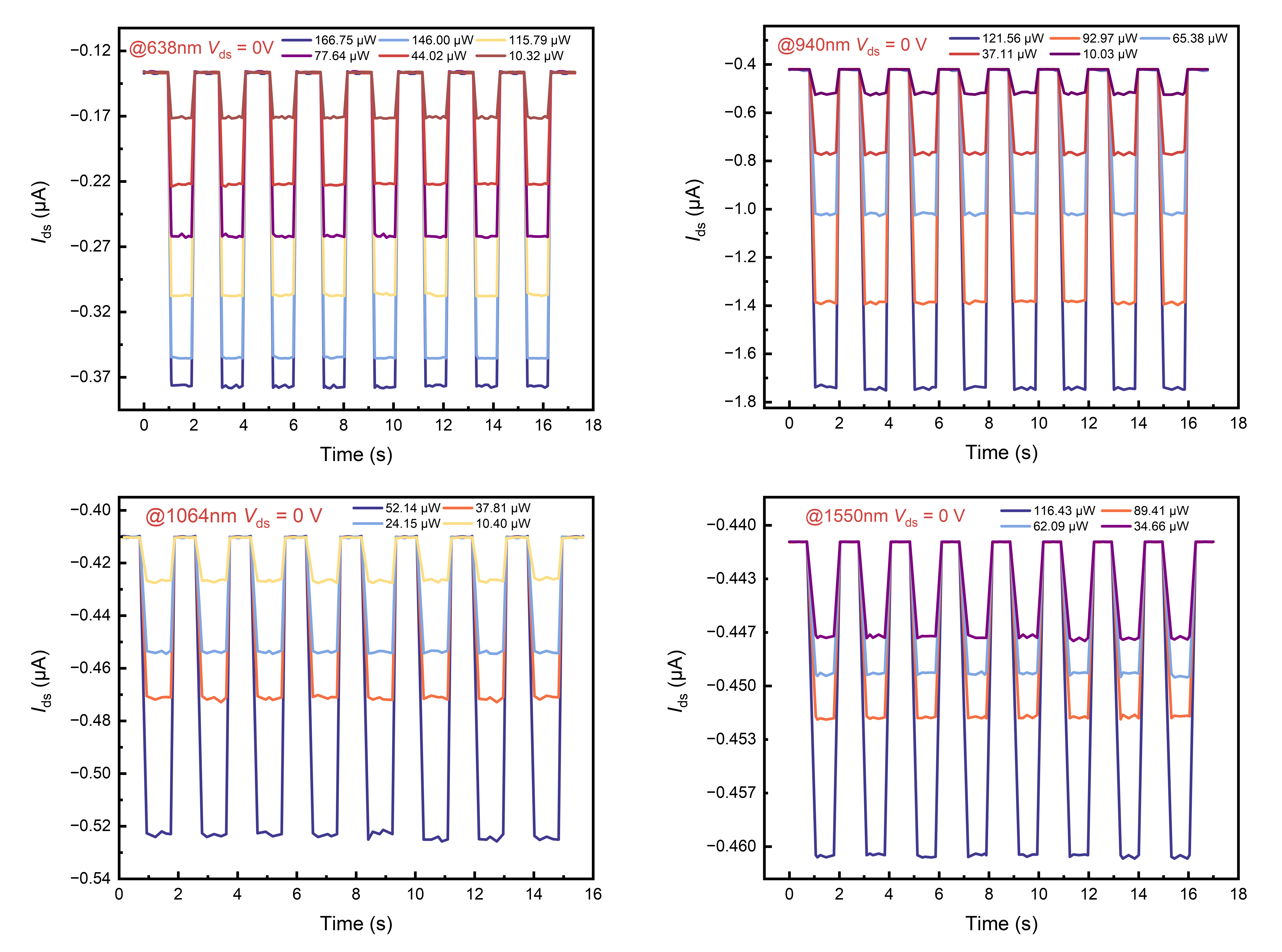


**Figure S6 The photocurrent switching characteristics of Ta_2_NiSe_5_/Sb_2_Te_3_ heterojunction photodetectors under 0V bias voltages in 638 nm to 1550 nm.** a-d) The temporal photoresponse of Ta_2_NiSe_5_/Sb_2_Te_3_ heterojunction photodetector from 638 nm to 1550 nm under various incident light powers. All measurements were carried out in ambient air tested by our source meter (Keithley 6482) at a modulation frequency of 0.5 Hz.

**7. The responsivity of Ta_2_NiSe_5_/Sb_2_Te_3_ photodetector varies with the incident light power.**

The photocurrent is extracted from Figure S5, and the responsivity under each power is calculated by the formula: *R*_A_ = *I*_ph_ / *P*_eff_. Based on the equation, the evaluation of the linear dynamic-stability is based on the variation of the responsivity, as shown in Figure S7. The responsivity curve, predominantly linear with minor fluctuations, validates the excellent linear dynamic stability of the photodetector.


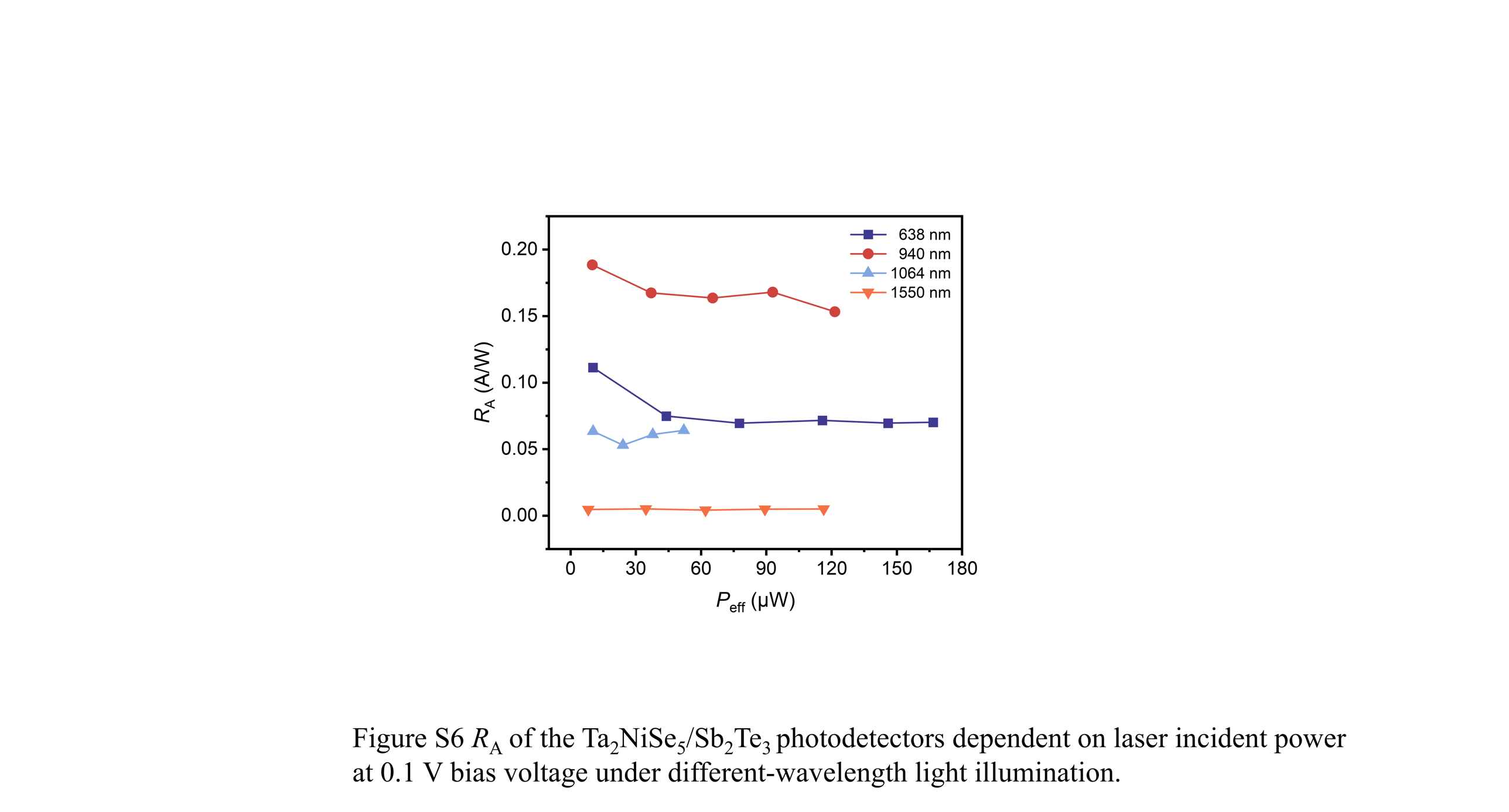


**Figure S7 *R*_A_ of the Ta_2_NiSe_5_/Sb_2_Te_3_ photodetectors dependent on laser incident power at 0.1 V bias voltage under 638 nm, 940 nm, 1064 nm, and 1550 nm illumination.**

**8. *D*^*^ and EQE of Ta_2_NiSe_5_/Sb_2_Te_3_ heterojunction devices**

*D*^*^ represents the detection ability of photodetectors for weak light. EQE reflects the quantum efficiency of photodetectors. By extracting the photocurrent of different incident light power, through the formula: *D*^*^ = (*A*·Δ*f* )^1/2^/NEP = (*A*·Δ*f* )^1/2^*R*_A_/*i*_n_, where *A* is the effective area of the device, Δ*f* = 1Hz, and the noise equivalent power can be expressed as NEP = *i*_n_/*R*_A_. EQE = *hcR*_A_/eλ, where *h* is 6.626×10^-34^, *c* is 3×10^8^ m·s^-1^, *e* is 1.6×10^-19^ C and *i*_n_ = (*i*_t_^2^ + *i*_s_^2^)^1/2^. Figure S8 a.b.c.d shows the trend of *D*^*^ and EQE of photodetectors with the change of incident light power.


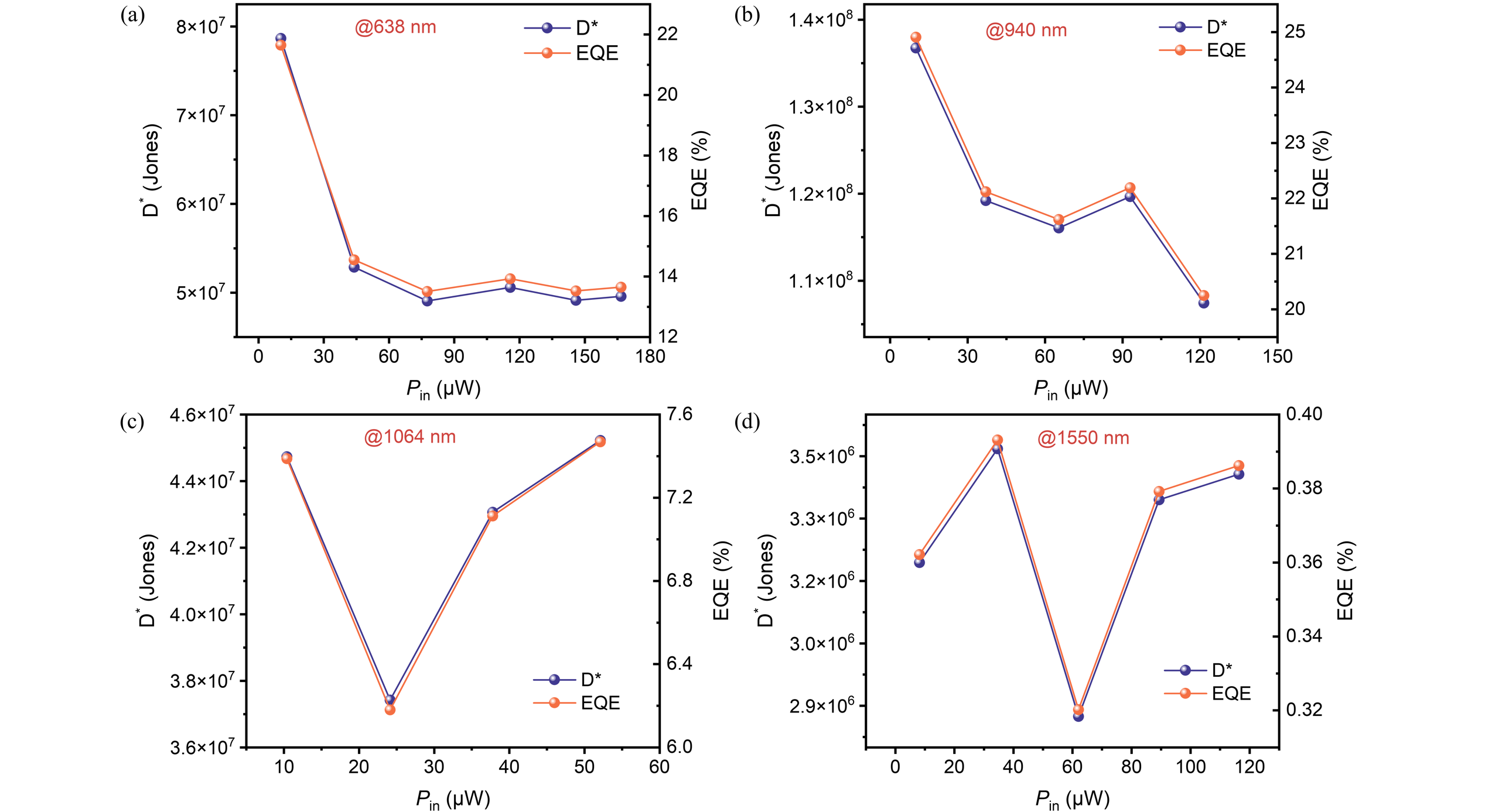


**Figure S8 *D*^*^ and EQE of Ta_2_NiSe_5_/Sb_2_Te_3_ heterojunction devices with the variation of incident light power at different wavelengths under 0.1 V bias voltage.** a-d) The *D*^*^ and EQE of Ta_2_NiSe_5_/Sb_2_Te_3_ heterojunction photodetector from 520 nm to 1650 nm under various incident light powers.

**9. The *V*_ds_-*I*_ds_ diagram of Ta_2_NiSe_5_/Sb_2_Te_3_ heterojunction photodetector under 0.1 THz illumination.**

Before the THz response testing, the I-V curve was measured. Figure S9 shows the output characteristic curve of Ta_2_NiSe_5_/Sb_2_Te_3_ photodetector at 0.1 THz, which showing a good ohmic contact.


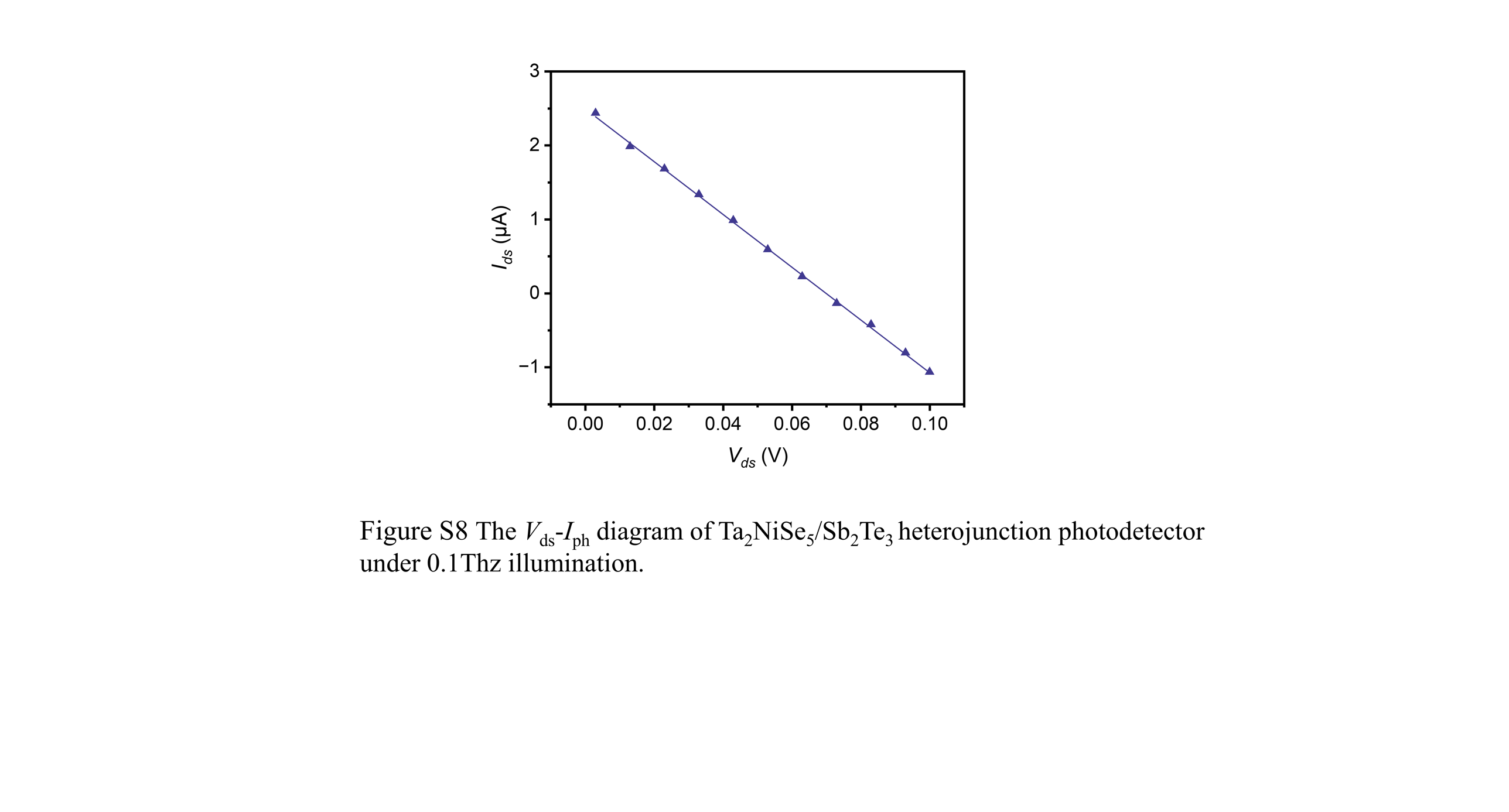


**Figure S9 The *V*_ds_-*I*_ds_ diagram of Ta_2_NiSe_5_/Sb_2_Te_3_ heterojunction photodetector under 0.1THz illumination.**

**10. Photocurrent of the Ta_2_NiSe_5_/Sb_2_Te_3_ photodetector at THz frequency.**

The THz source is generated from a microwave source equipped with a Virginia Diodes Inc. (VDI) frequency multiplier chain, operating in the frequency range of 0.02-0.13 THz. As shown in Figure S10, the heterojunction photodetector exhibits a significant photocurrent response at -0.1V bias.

**Figure S10 Photocurrent of the Ta_2_NiSe_5_/Sb_2_Te_3_ photodetector at a frequency from 0.02 to 0.13 THz at *V*_ds_ = -0.1 V.**

**11. Profiles across the carrier *T*(*x*), *E*_F_(*x*), *S*(*x*), and potential gradient based on heterojunction and butterfly junction electrodes at different bias voltage.**


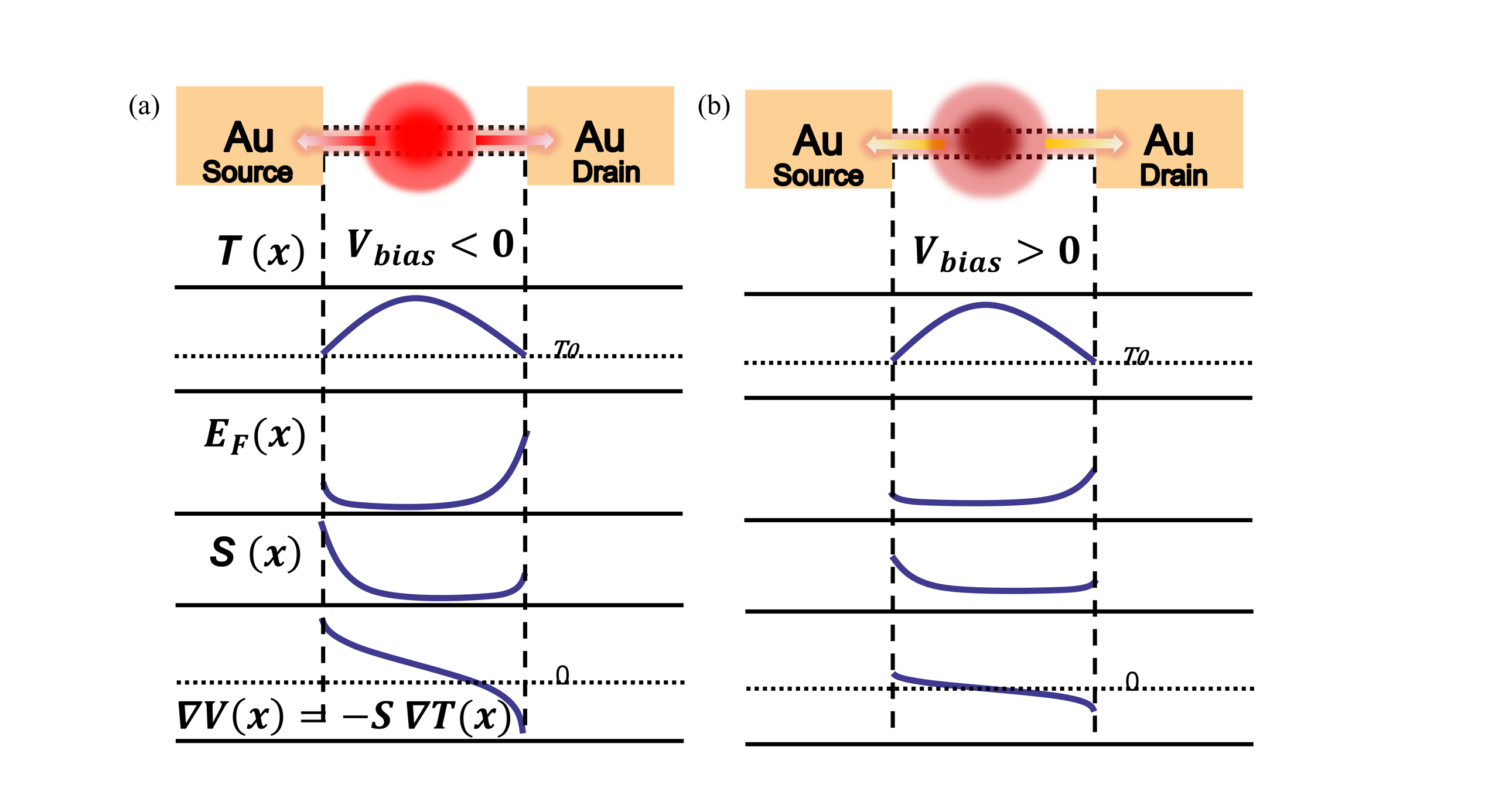


Figure S11 Illustration of the band diagram of the Ta_2_NiSe_5_/Sb_2_Te_3_ photodetector, and profiles across the carrier temperature $T(x)$, Fermi energy $E_{F}(x)$, Seebeck coefficient $S(x)$, and potential gradient $\nabla V(x)$based $\nabla V\left( x \right)$ on heterojunction at negative bias voltage (a) and positive bias voltage (b). The photoresponse is proportional to the integral of$\nabla V\left( x \right)$over the channel of the device.

**12. Photocurrent versus varying modulated frequency in the THz region.**

3dB bandwidth refers to the corresponding bandwidth when the signal power is reduced to half of the maximum value, this often used to evaluate the system frequency response characteristics and bandwidth limitations. Figure S12 shows the photocurrent versus the modulation frequency with ∼10 kHz designated as a 3 dB frequency.


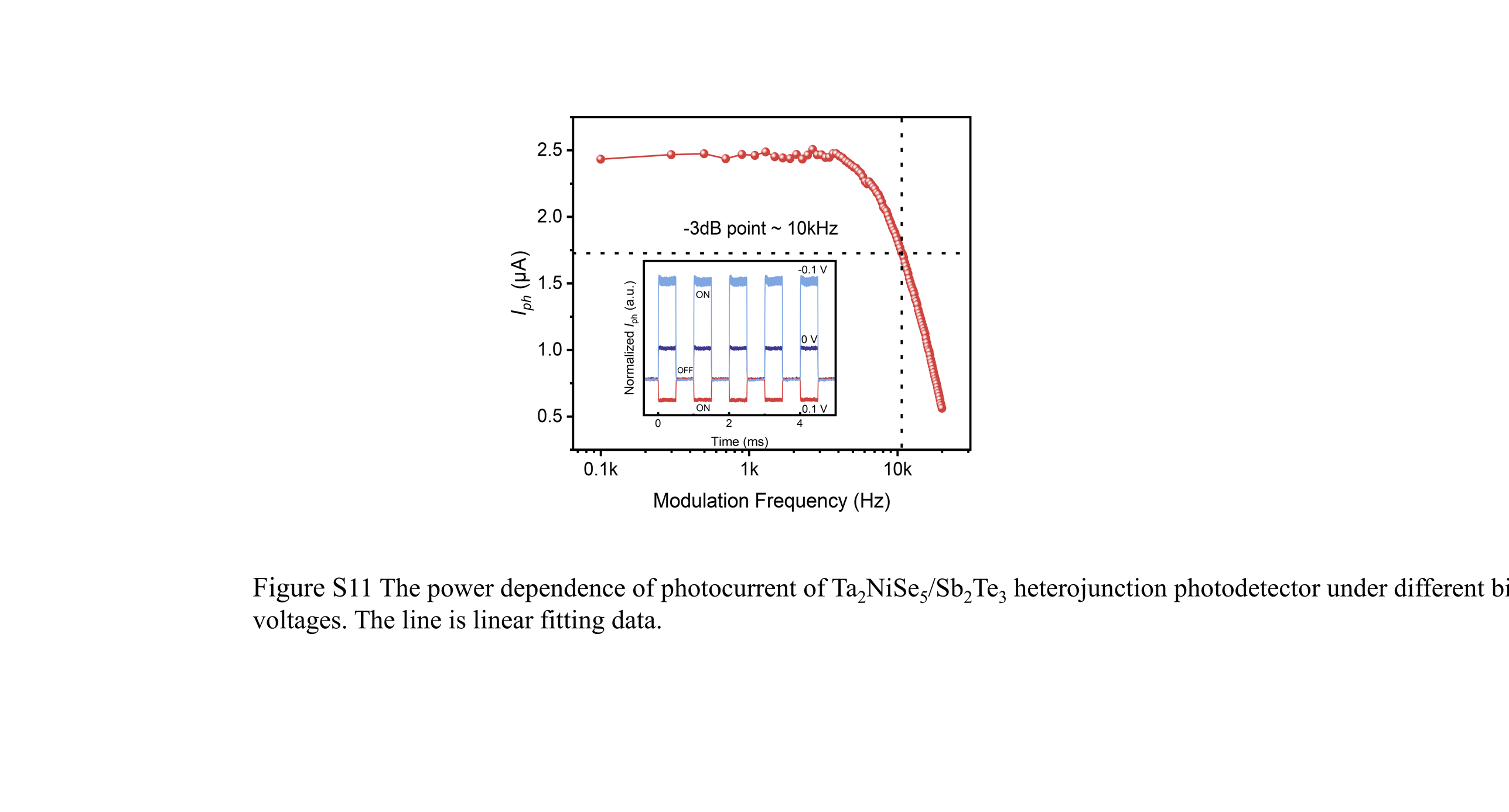


**Figure S12** **Photocurrent versus varying modulated frequency, indicating a 3 dB frequency of ∼10 kHz.** The embedded graph is the photoresponse waveform of the PD under 0.10 THz illumination with different bias voltage (-0.1, 0, and 0.1 V).
